# Impact of heterozygous ALK1 mutations on the transcriptomic response to BMP9 and BMP10 in endothelial cells from hereditary hemorrhagic telangiectasia and pulmonary arterial hypertension donors

**DOI:** 10.1007/s10456-023-09902-8

**Published:** 2024-01-31

**Authors:** T. Al Tabosh, H. Liu, D. Koça, M. Al Tarrass, L. Tu, S. Giraud, L. Delagrange, M. Beaudoin, S. Rivière, V. Grobost, M. Rondeau-Lutz, O. Dupuis, N. Ricard, E. Tillet, P. Machillot, A. Salomon, C. Picart, C. Battail, S. Dupuis-Girod, C. Guignabert, A. Desroches-Castan, S. Bailly

**Affiliations:** 1 Biosanté unit U1292, Grenoble Alpes University, INSERM, CEA, 38000 Grenoble, France; 2 Faculté de Médecine, Pulmonary Hypertension: Pathophysiology and Novel Therapies, Université Paris-Saclay, 94276 Le Kremlin-Bicêtre, France; 3 INSERM UMR_S 999 «Pulmonary Hypertension: Pathophysiology and Novel Therapies», Hôpital Marie Lannelongue, 92350 Le Plessis-Robinson, France; 4 Genetics Department, Femme-Mère-Enfants Hospital, Hospices Civils de Lyon, 69677 Bron, France; 5 National Reference Center for HHT, 69677 Bron, France; 6 Internal Medicine Department, CHU of Montpellier, St Eloi Hospital and Center of Clinical Investigation, INSERM, CIC 1411, 34295 Montpellier Cedex 7, France; 7 Internal Medicine Department, CHU Estaing, 63100 Clermont-Ferrand, France; 8 Internal Medicine Department, University Hospital of Strasbourg, 67091 Strasbourg Cedex, France; 9 Hôpital Lyon SUD, Hospices Civils de Lyon, Université Claude Bernard Lyon 1, 69100 Villeurbanne, France; 10 Faculty of Medicine, Lyon University, 69921 Lyon, France

**Keywords:** ALK1, BMP, HHT, PAH, RNA-seq, LFNG

## Abstract

Heterozygous activin receptor-like kinase 1 (*ALK1*) mutations are associated with two vascular diseases: hereditary hemorrhagic telangiectasia (HHT) and more rarely pulmonary arterial hypertension (PAH). Here, we aimed to understand the impact of *ALK1* mutations on BMP9 and BMP10 transcriptomic responses in endothelial cells. Endothelial colony-forming cells (ECFCs) and microvascular endothelial cells (HMVECs) carrying loss of function *ALK1* mutations were isolated from newborn HHT and adult PAH donors, respectively. RNA-sequencing was performed on each type of cells compared to controls following an 18 h stimulation with BMP9 or BMP10. In control ECFCs, BMP9 and BMP10 stimulations induced similar transcriptomic responses with around 800 differentially expressed genes (DEGs). *ALK1*-mutated ECFCs unexpectedly revealed highly similar transcriptomic profiles to controls, both at the baseline and upon stimulation, and normal activation of Smad1/5 that could not be explained by a compensation in cell-surface ALK1 level. Conversely, PAH HMVECs revealed strong transcriptional dysregulations compared to controls with > 1200 DEGs at the baseline. Consequently, because our study involved two variables, ALK1 genotype and BMP stimulation, we performed two-factor differential expression analysis and identified 44 BMP9-dysregulated genes in mutated HMVECs, but none in ECFCs. Yet, the impaired regulation of at least one hit, namely lunatic fringe (*LFNG*), was validated by RT-qPCR in three different *ALK1*-mutated endothelial models. In conclusion, *ALK1* heterozygosity only modified the BMP9/BMP10 regulation of few genes, including *LFNG* involved in NOTCH signaling. Future studies will uncover whether dysregulations in such hits are enough to promote HHT/PAH pathogenesis, making them potential therapeutic targets, or if second hits are necessary.

**Supplementary Information:**

The online version contains supplementary material available at 10.1007/s10456-023-09902-8.

## Introduction

Hereditary hemorrhagic telangiectasia [HHT (MIM: 187300, 600376, 175050)], also known as Osler–Weber–Rendu syndrome, is a rare genetic vascular disease characterized by the development of multiple focal vascular malformations (VMs), including muco-cutaneous telangiectases responsible for recurrent, spontaneous epistaxis and visceral arteriovenous malformations [1]. HHT is an autosomal-dominant disease presenting complete penetrance over the age of 50, but with highly variable expressivity [1]. It is mostly caused by mutations in *ENG* (MIM: 131195) and *ACVRL1* (MIM: 601284), which account for 85% of cases, and more rarely in *SMAD4* (MIM: 600993) accounting for less than 2% [2, 3]. Additionally, mutations in *GDF2* (MIM: 615506) were reported in few individuals presenting HHT-like symptoms [4]. The protein products of all four genes are components of the BMP/TGFß signaling pathway. *ACVRL1* (hereafter referred to as *ALK1*) encodes for the type I receptor ALK1, *ENG* for the co-receptor endoglin, *SMAD4* for a transcription factor downstream of the receptors and *GDF2* for BMP9, a high affinity ligand for this receptor complex [5]. The current signaling model is that a BMP9 or BMP10 dimer binds to the co-receptor endoglin, which facilitates their binding to the signaling complex composed of the type I receptor ALK1 and a type II receptor that can either be BMPRII, ActRIIB or ActRIIA. Upon BMP9 or BMP10 binding, the type II receptor phosphorylates ALK1, which subsequently phosphorylates the transcription factors Smad1, Smad5 and Smad8, allowing their interactaction with Smad4. The formed trimeric Smad complex then translocates to the nucleus where it binds to DNA together with other transcription factors to regulate the expression of many genes [6]. The receptor ALK1 and its co-receptor endoglin are mostly expressed on endothelial cells (ECs), supporting a key role for these receptors in vascular physiology [7]. It has been demonstrated that the BMP9/BMP10-ALK1-endoglin signaling pathway promotes vascular quiescence [8]. The current working model is that mutations within this signaling pathway would lead to an increase in angiogenesis. Thus, anti-angiogenic drugs, mainly blocking the VEGF signaling pathway, have been proposed as a therapeutic option for HHT patients and tend to improve symptoms (bleedings and high cardiac output secondary to liver AVMs) but do not cure the disease [9, 10].

HHT causal mutations result in the loss of function (LOF) of the gene product [11, 12] and are inherited through an autosomal-dominant manner. Therefore, HHT has long been speculated to be caused by haploinsufficiency of the mutated *ENG* or *ALK1* gene product, with previous reports pointing at a reduction in endoglin and ALK1 expression in HHT1 and HHT2 patient-derived cells, respectively [13]. However, haploinsufficiency does not explain why vascular lesions in HHT patients occur focally in specific vascular beds [14], despite the systemic presence of the pathogenic germline mutation in all blood vessels.

Intriguingly, mutations in the same signaling pathway have been identified in rare cases of pulmonary arterial hypertension (PAH) [15]. PAH is defined by a resting mean pulmonary artery pressure (mPAP) > 20 mmHg with a pulmonary artery wedge pressure ≤ 15 mmHg and elevated pulmonary vascular resistance [(PVR) of > 2 Wood units] [16, 17]. PAH can develop sporadically, in association with risk factors such as certain drugs or other diseases, or can be inherited. Although *BMPR2* mutations are major predisposing factors for idiopathic (15–40%) and heritable (60–80%) PAH, less common or rare mutations in other genes encoding key members of the Smad1/5/8 signaling pathway, including *ALK1*, *GDF2* (BMP9) and *BMP10* have been identified, underlining the critical role of this pathway in PAH [15].

*ALK1* mutations are responsible for nearly half HHT cases and a minority of PAH patients, and the same *ALK1* mutations can predispose to any of the two vascular disorders [18–20]. Despite our understanding of the genetics and of downstream pathways involved in HHT and PAH, the molecular mechanisms that initiate HHT-related VMs and PAH are poorly understood [21]. Here, we aimed to understand the impact of *ALK1* mutations on the downstream BMP9/BMP10-ALK1 signaling pathway by studying the transcriptome of heterozygote *ALK1*-mutated primary ECs to better understand the underlying molecular mechanisms and to propose new therapeutic approaches for these two diseases. To address this question, we used two different endothelial models carrying heterozygous LOF *ALK1* mutations: (1) endothelial colony-forming cells (ECFCs) that were isolated from the cord blood of newborns with an HHT-affected parent and (2) Human microvascular endothelial cells (HMVECs) isolated from explanted lungs of PAH patients.

We first used ECFCs from newborns, which allowed us to address the sole role of heterozygous *ALK1* mutations in cells probably not yet exposed to a sick microenvironment. These circulating cells, possessing vasoregenerative potential [22], are a particularly interesting model as they could potentially be involved in EC turnover in the liver leading to the reported recurrence of hepatic VMs in a subset of HHT patients receiving a liver transplantation [23]. We also had access to *ALK1*-mutated microvascular endothelial cells (HMVECs) from the explanted lungs of two PAH patients, which is a very rare event as *ALK1* mutations occur in only around 6% of patients with heritable PAH. In turn, heritable cases do not exceed 10% of all PAH patients, which are estimated as 15–50 individuals per million [15]. The limited availability of *ALK1*-mutated HMVECs prompted us to additionally include HMVECs from 3 PAH patients with more frequent heterozygous mutations in the type II BMP9/10 receptor *BMPR2*, for validation of target genes by RT-qPCR. In order to validate some of the results, we also used human umbilical vacular endothelial cells (HUVECs) derived from the umbilical cords of newborns with an HHT-affected parent.

We performed RNA-sequencing on each type of cells compared to control counterparts, either nonstimulated or following an overnight stimulation with BMP9 or BMP10. Together, our data support that *ALK1* heterozygosity does not impact the activation of the canonical Smad pathway by BMP9/10, nor cause overt transcriptional dysregulations in ECFCs. However, PAH HMVECs revealed strong transcriptional dysregulations compared to controls both at the baseline and in the presence of BMPs. Thus, in order to take into account the two variables (*ALK1* mutation and stimulation), we performed two-factor differential expression analysis on HMVECs’ RNA-seq datasets that highlighted few genes exhibiting impaired regulation by BMP9/BMP10 in mutated cells. These included *LFNG*, encoding the NOTCH signaling modulator lunatic fringe, which was also found differentially regulated in *ALK1*-mutated ECFCs and HUVECs and might thus be important in the pathogenesis of HHT and PAH.

## Results

### Isolation and characterization of ECFCs carrying ALK1 mutations

**Fig. 1:**
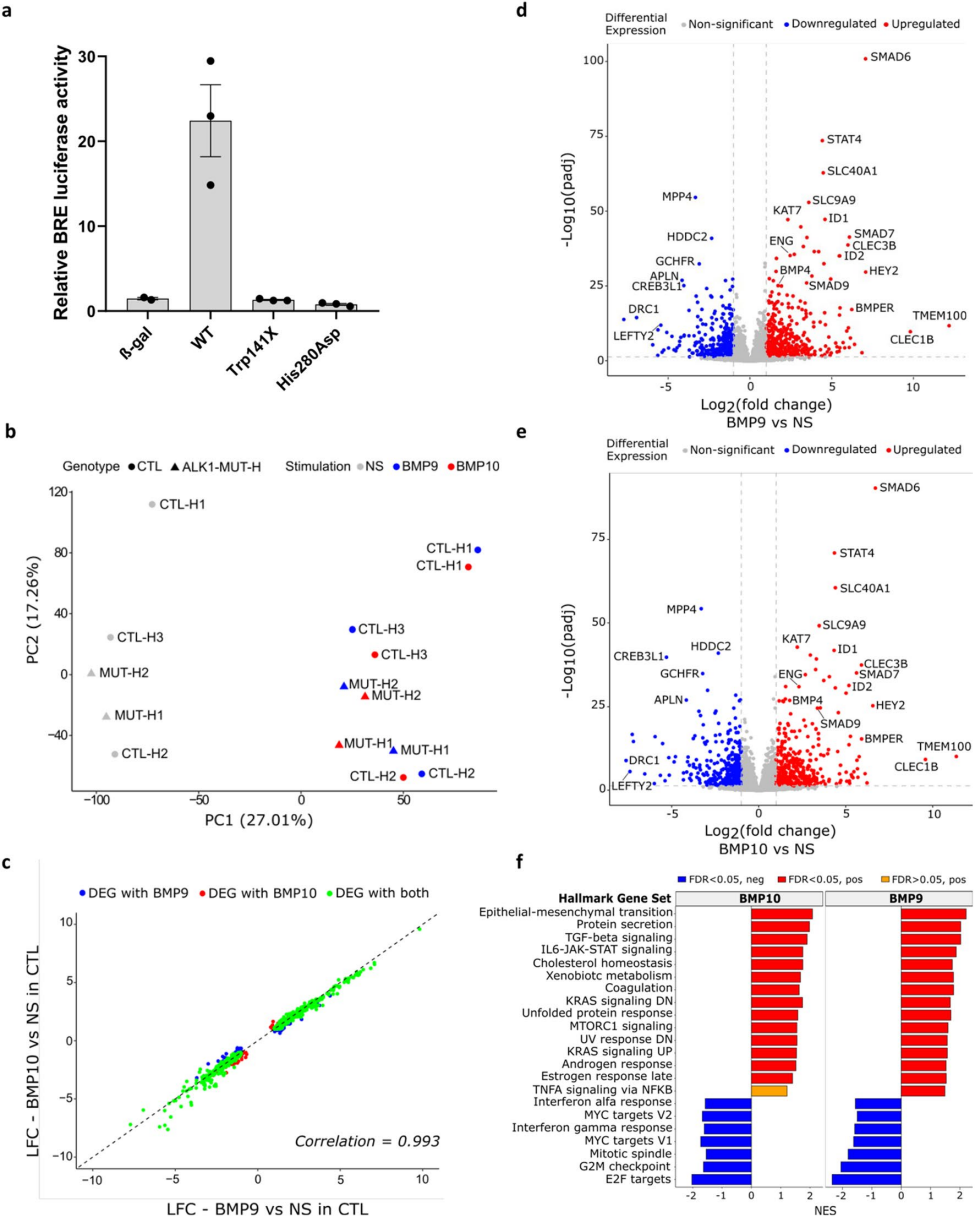
BMP9 and BMP10 induce a similar transcriptomic response in control ECFCs. **a** Relative BMP Response Element (BRE) luciferase activity measured in NIH-3T3 cells overexpressing either WT or mutant *ALK1* plasmids identified in *ALK1*-mutated ECFCs (p.Trp141X, MUT-H1) and (p.His280Asp, MUT-H2) used in the ECFC RNA-seq analysis. BRE firefly luciferase activities were normalized to renilla luciferase activity. Data shown are mean ± SEM from three independent experiments. **b**–**f** 3 CTL (CTL-H1, CTL-H2 and CTL-H3) and 2 *ALK1*-mutated ECFCs (MUT-H1 and MUT-H2) were stimulated or not with BMP9 or BMP10 (10 ng/mL) for 18 h. The experiment was repeated three times after which bulk RNA-seq analysis was performed. **b** Principal component analysis (PCA) showing clustering of RNA-seq samples by treatment (BMP9 or BMP10 stimulation vs NS) in CTL and MUT ECFCs. Each dot represents the mean of three experiments for one sample. **c** Scatter plot comparing log_2_ fold change (LFC) values of protein coding DEGs regulated in CTL ECFCs by BMP9 vs those regulated by BMP10 both compared to NS. Pearson correlation is reported. **d**, **e** Volcano plot representations showing global changes in gene expression in CTL ECFCs after BMP9 (**d**) or BMP10 (**e**) stimulation. DEGs with high LFC and high statistical significance are annotated. **f** Gene-set enrichment analysis (GSEA) performed using hallmark gene sets. The bar plot represents the top significant gene set categories enriched in CTL ECFCs upon BMP10 or BMP9-stimulation ordered using normalized enrichment scores (NES)

Control (CTL-H) and *ALK1*-mutated ECFCs (MUT-H) from newborns who have inherited a heterozygous *ALK1* mutation from an HHT parent were clonally isolated from cord blood following the recommendations of the Vascular Biology Standardization Subcommittee [24]. Isolated ECFCs (3 CTL-H and 2 MUT-H, Table 1) displayed the classical endothelial cobblestone-like morphology (Fig. S1a) and were VE-cadherin (CD144) positive (Fig. S1b, c). These cells were also positive for the EC markers CD31 and CD146 and negative for the hematopoietic cell-specific surface antigen CD45 (Fig. S1d–f), confirming their endothelial identity. The functional activity of the two ALK1 mutants (MUT-H1; non sense mutation p.Trp141X and MUT-H2; missense mutation p.His280Asp, Table 1) was tested using the BMP response element (BRE) luciferase reporter assay in cells transfected with equal amounts of plasmids encoding either WT or mutated ALK1 (Fig. 1a), as previously described by our group [12]. Unlike cells transfected with wildtype *ALK1*, those exogenously expressing either of the two ALK1 mutants were unable to respond to a BMP9 stimulation (100 pg/mL, Fig. 1a), supporting that both studied mutations lead to loss of function.

**Table 1:** List of ALK1-mutated ECFCs and HUVECs isolated from HHT donors

ID	ALK1 mutation		Type of mutation	Domain	Cells isolated	Assays
MUT-H1	c.423G>A	p.Trp141X	Nonsense	Transmembrane	ECFC	RNA-seq, RT-qPCR, p-Smad1/5 IF, BLA, FC
MUT-H2	c.838C>G	p.His280Asp	Missense	Kinase	ECFC	RNA-seq, RT-qPCR, p-Smad1/5 IF, BLA, FC
MUT-H3	c.190C>T	p.Gln64X	Nonsense	Extracellular	ECFC/HUVEC	RT-qPCR (ECFC and HUVEC), p-Smad1/5 IF, BLA, FC
MUT-H4	c.1231C>T	p.Arg411Trp	Missense	Kinase	ECFC/HUVEC	RT-qPCR (ECFC and HUVEC), p-Smad1/5 IF, BLA, FC
MUT-H5	c.1112dup	p.Thr372HisfsX20	Frameshift	Kinase	ECFC/HUVEC	RT-qPCR (ECFC and HUVEC), FC
MUT-H6	c.1413C>G	p.Cys471Trp	Missense	Kinase	ECFC/HUVEC	BLA, FC
CTL-H1	–	–	–	–	ECFC	RNA-seq, RT-qPCR, BLA, FC
CTL-H2	–	–	–	–	ECFC	RNA-seq, RT-qPCR, p-Smad1/5 IF, BLA, FC
CTL-H3	–	–	–	–	ECFC	RNA-seq, RT-qPCR, p-Smad1/5 IF, BLA, FC
CTL-H4	–	–	–	–	ECFC/HUVEC	RT-qPCR (ECFC and HUVEC), p-Smad1/5 IF, BLA, FC
CTL-H5	–	–	–	–	HUVEC	RT-qPCR
CTL-H6	–	–	–	–	HUVEC	RT-qPCR

List of CTL and *ALK1*-mutated ECFCs and HUVECs that were isolated from newborns indicating the carried mutation (DNA and protein), the type of mutation, the mutated ALK1 domain and the assays in which they were used

*RNA-seq* RNA-sequencing, *RT-qPCR* reverse transcription quantitative polymerase chain reaction, *p-Smad1/5 IF* phospho-Smad1/5 immunofluorescence, *BLA* (BRE) BMP responsive element luciferase assay, *FC* flow cytometry

### RNA-seq analysis in CTL ECFCs in response to BMP9 or BMP10 stimulation

In order to decipher the impact of *ALK1*-mutations on gene regulation in response to BMP9 and BMP10, RNA-sequencing was performed on ECFCs from 3 CTLs (CTL-H1–3) and 2 *ALK1* mutated ECFCs (MUT-H1–2) (Table 1) that were either nonstimulated (NS) or stimulated with BMP9 or BMP10. To facilitate the detection of a high number of regulated targets and to better mimic physiological conditions, where circulating BMP9 and BMP10 are continuously replenished, cells were stimulated with BMP9 or BMP10 for a prolonged duration of 18 h. In line with that, as depicted in Fig. S2a, the mRNA expression of the BMP target *ID1* increased with time (1–18 h) in response to BMP9 stimulation (10 ng/mL). Subsequently, having chosen to study gene regulation following a prolonged BMP9 or BMP10 stimulation, a relatively high BMP dose was required, as even the strong BMP target *ID1* displayed significant upregulation only at 10 ng/mL in ECFCs (Fig. S2b). The experiment was repeated three times, after which RNA extraction for all samples was performed, followed by quality control, library preparation and RNA sequencing. Principal component analysis (PCA) could discriminate stimulated (BMP9 or BMP10, blue and red shapes, respectively) from NS conditions (grey shape) in all ECFCs but could not discriminate CTL ECFCs from *ALK1*-mutated ECFCs (Fig. 1b).

We first analyzed the BMP9 and BMP10 response versus (vs) NS condition in CTL ECFCs. Differential gene expression analysis using an absolute log_2_ fold change (LFC) threshold of 1 (lLFC(BMP response/NS condition)l ≥ 1) and an adjusted *p*-value (*p*adj) ≤ 0.05 (Benjamini–Hochberg procedure; Supplementary information SI1) identified respectively 828 and 787 protein-coding differentially expressed genes (DEGs) upon BMP9 or BMP10 stimulation (Table 2). These DEGs were nearly equally distributed between up and down-regulated genes (Table 2). Interestingly, BMP9 and BMP10 induced a highly similar transcriptomic response evidenced by the high Pearson correlation coefficient of 0.993 when plotting the LFCs of the DEGs obtained in response to BMP9 vs those obtained in response to BMP10 (Fig. 1c) and the high number of shared DEGs regulated by both ligands (81.4%; Fig. S3a). This similarity was also reflected in the volcano plots of BMP9- and BMP10-stimulated vs NS ECFCs, which highlighted the same top dysregulated genes in terms of LFC or padj (Fig. 1d, e). Among these top DEGs, we detected many genes known to be regulated by BMP9 and BMP10 in other ECs (*SMAD6*, *SMAD7*, *SMAD9*, *ID1*, *ID2*, *ENG*, *HEY2*, *BMPER*, *TMEM100*, *APLN*) (Fig. 1d, e). We also identified new DEGs with very similar regulation patterns between BMP9 and BMP10, including *STAT4*, *SLC40A1*, *SLC9A9*, *CLEC1B*, *CLEC3B*, *BMP4*, *KAT7*, *MPP4*, *HDDC2*, *CREB3L1*, *DRC1*, *GCHFR*, and *LEFTY2* (Fig. 1d, e). In accordance with the high similarity between BMP9 and BMP10 response, no DEGs could be identified when directly comparing BMP9 to BMP10-stimulated CTL ECFCs (Table 2). Additionally, gene set enrichment analysis (GSEA) using hallmark gene sets from MsigDB was performed independently on total genes (whether DEGs or not) regulated by BMP9 or BMP10 in CTL ECFCs (Supplementary information SI2). The top 20 enriched genesets identified were very similar between BMP9 and BMP10 (Fig. 1f). As expected, TGFß signaling was positively enriched by BMP9 and BMP10 stimulation, but we could also identify epithelial-mesenchymal transition, protein secretion and several signaling pathways (IL6-JAK-STAT3, KRAS and MTORC1) as positively enriched terms (Fig. 1f). On the other hand, many hallmarks related to cell cycle (MYC-targets, mitotic spindle, G2M checkpoint and E2F targets) were negatively enriched (Fig. 1f), supporting the reported role of BMP9 and BMP10 in maintaining vascular quiescence [8, 25]. Together, these results demonstrate that under these stimulatory conditions, BMP9 and BMP10 induce a very similar transcriptomic response in CTL ECFCs.

**Table 2:** Number of protein-coding DEGs identified by differential expression analysis in CTL and ALK1-mutated ECFCs and HMVECs

Comparison	ECFCs			HMVECs		
	Total DEGs	Upregulated	Downregulated	Total DEGs	Upregulated	Downregulated
CTL NS vs B9	828	456	372	704	366	338
CTL NS vs B10	787	418	369	481	225	256
CTL B9 vs B10	0	0	0	2	0	2
MUT NS vs B9	604	310	294	295	158	137
MUT NS vs B10	564	278	286	206	90	116
MUT B9 vs B10	0	0	0	0	0	0
CTL NS vs MUT NS	28	11	17	1261	528	733
CTL B9 vs MUT B9	19	6	13	1262	502	760
CTL B10 vs MUT B10	30	7	23	1164	503	661

3 CTL ECFCs and 2 *ALK1*-mutated ECFCs (MUT-H1–H2) or 3 CTL HMVECs and 2 *ALK1*-mutated HMVECs (MUT-P1–P2) were stimulated or not with BMP9 or BMP10 (10 ng/mL) for 18 h. The experiment was repeated three times to generate technical replicates, after which bulk RNA-seq analysis was performed using DESeq2 package with an absolute log_2_ fold change threshold of 1 (llog_2_FCl ≥ 1) and a adjusted *p*-value ≤ 0.05 (Benjamini–Hochberg procedure). Data show protein coding DEGS analyzed by *NS* non-stimulated, *B9* BMP9-stimulated, *B10* BMP10-stimulated

### *ALK1* heterozygosity in ECFCs does not impair the global transcriptomic response to BMP9 or BMP10

To uncover the effect of heterozygous *ALK1* mutations on gene regulation, we analyzed the basal transcriptome of MUT versus CTL ECFCs, before any stimulation, and in response to BMP9 or BMP10. In accordance with the PCA, which could not differentiate CTL from MUT ECFCs (Fig. 1b), only 28 DEGs were identified between NS CTL and NS MUT ECFCs (Table 2). Similarly, following a BMP9 or BMP10 stimulation, only 19 and 30 DEGs, respectively, were significantly differentially expressed between the two ECFC groups (Table 2).

Upon analyzing the transcriptomic response of MUT ECFCs to BMP9 or BMP10, 604 and 564 DEGs were identified, respectively (Table 2). As in CTL ECFCs, BMP9 and BMP10 induced a similar response in *ALK1*-mutated cells, with 77.2% DEGs commonly regulated by both ligands (Fig. S3b) and a highly similar global regulation pattern (Pearson correlation coefficient = 0.989, Fig. S3c).

**Fig. 2:**
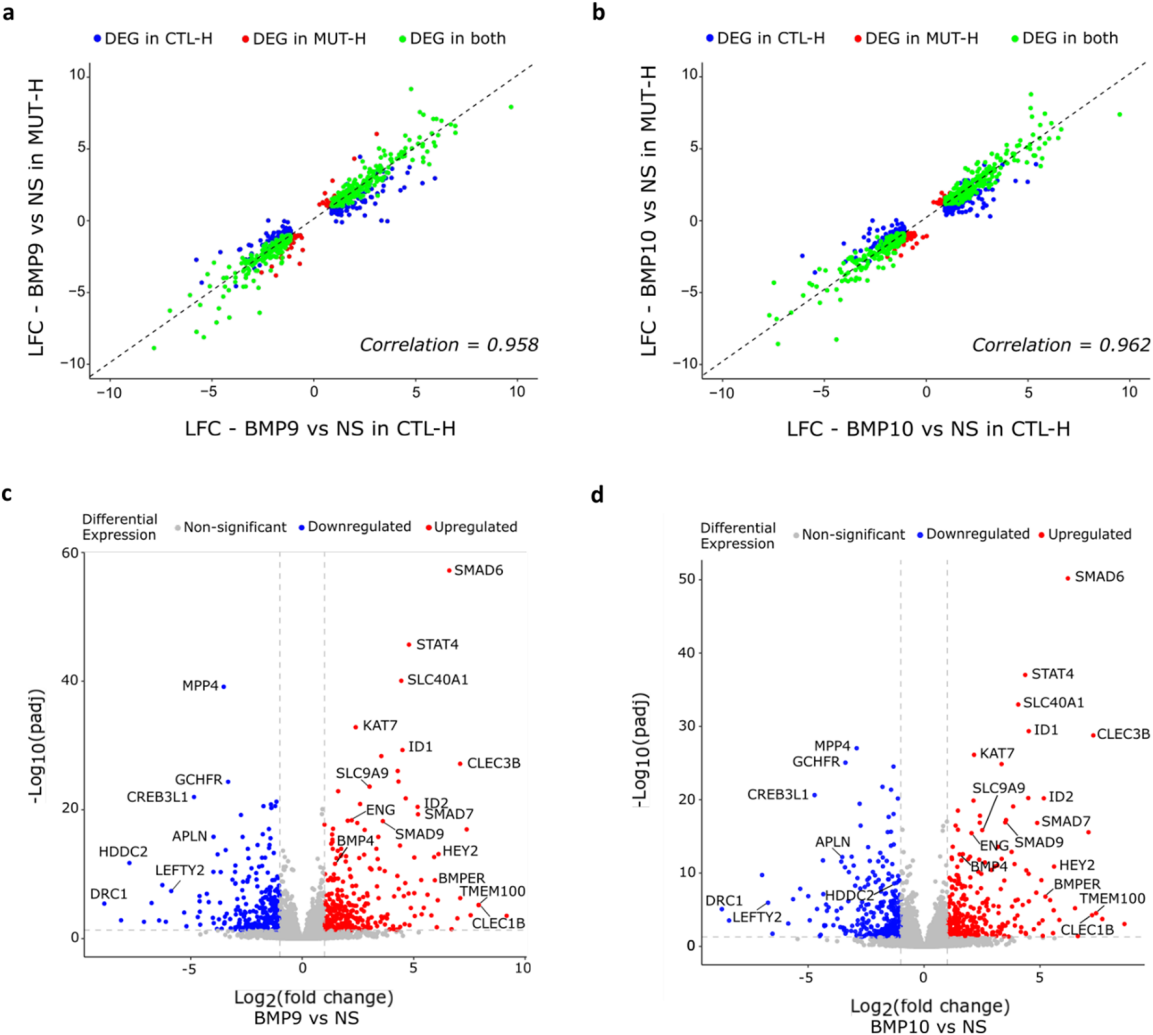
ALK1 heterozygosity in ECFCs does not impair the global transcriptomic response to BMP9 or BMP10. **a**–**d** 3 CTL (CTL-H1, CTL-H2 and CTL-H3) and 2 *ALK1*-mutated ECFCs (MUT-H1 and MUT-H2) were stimulated or not with BMP9 or BMP10 (10 ng/mL) for 18 h. The experiment was repeated three times after which bulk RNA-seq analysis was performed. **a**, **b** Scatter plots comparing log_2_ fold change (LFCs) values of protein coding DEGs regulated by BMP9 (**a**) or BMP10 (**b**) in CTL ECFCs vs MUT ECFCs. Pearson correlation is reported. **c**, **d** Volcano plots representations showing global changes in gene expression in *ALK1*-mutated ECFCs after BMP9 (**c**) or BMP10 (**d**) stimulation vs NS. DEGs with high LFC and high statistical significance are annotated

We next compared the LFCs of each list of target genes in stimulated CTL vs stimulated *ALK1*-mutated ECFCs and found that most regulated genes demonstrated the same regulation patterns in the two cell groups upon BMP9 or BMP10 stimulation (Pearson correlation coefficient = 0.958 and 0.962 respectively, Fig. 2a, b). Consistently, we could identify in *ALK1*-mutated ECFCs the same top protein-coding DEGs upon BMP9 or BMP10 stimulation as in CTL ECFCs (*SMAD6*, *SMAD7*, *SMAD9*, *ID1*, *ID2*, *ENG*, *HEY2*, *BMPER*, *TMEM100*, *STAT4*, *APLN*, *SLC40A1*, *SLC9A9*, *CLEC1B*, *CLEC3B*, *BMP4*, *KAT7*, *MPP4*, *HDDC2*, *CREB3L1*, *DRC1*, *GCHFR*, *LEFTY2*; Fig. 2c, d). Altogether, these data unexpectedly show that CTL and *ALK1*-mutated ECFCs display highly similar transcriptomic profiles following BMP9 or BMP10 stimulation.

### *ALK1* heterozygosity in ECFCs does not impair p-Smad1/5 response to BMP9

**Fig. 3:**
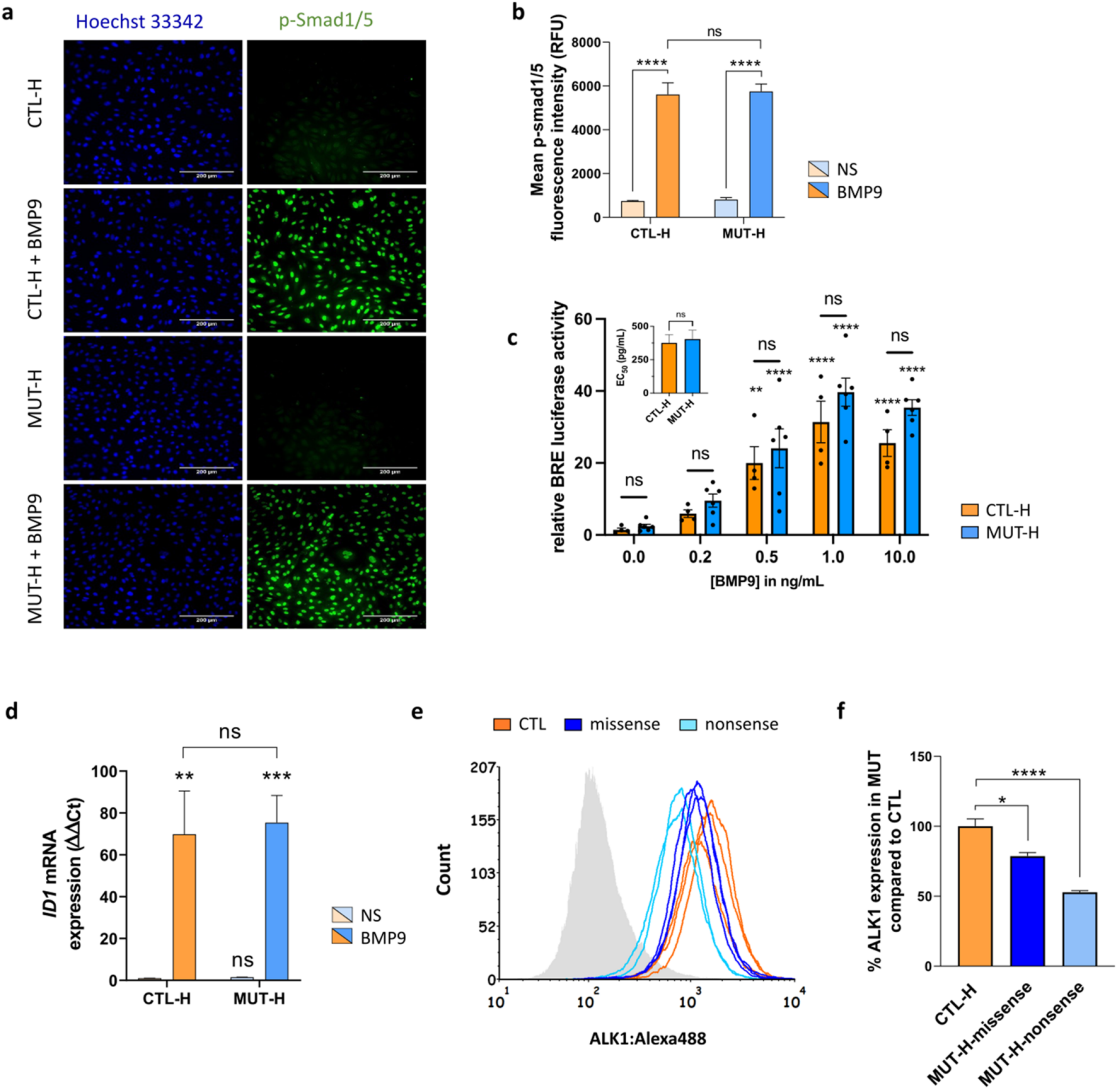
*ALK1* heterozygosity in ECFCs does not impair the p-Smad1/5 response to BMP9. **a**, **b** 3 CTL and 6 MUT ECFCs (MUT-H1–H6) were stimulated with BMP9 (10 ng/mL) for 1 h then fixed and immunostained for phospho-Smad1/5 (p-Smad1/5). Cells were stimulated in duplicates and at least 16 different fields were imaged in each well. **a** Representative p-Smad1/5 immunostainings in 1 CTL and 1 MUT ECFC in the absence or presence of BMP9 for 1 h. The nuclei were counterstained using Hoechst 33342. **b** p-Smad1/5 fluorescence was quantified in the nuclei using IN Carta Image Analysis Software. Data presented are mean relative fluorescent intensity (RFU) ± SEM of three independent experiments. **c** 4 CTL and 6 MUT (MUT-H1–6) ECFCs were transiently transfected with pGL3(BRE)2-luc and pRL-TK-luc. Cells were then either non-treated or stimulated with increasing concentrations of BMP9 (0.2, 0.5, 1, 10 ng/mL) for 6 h. Firefly luciferase activities were normalized to renilla luciferase activities. Data shown are mean ± SD from 1 representative experiment of 4, and each point corresponds to one donor. The inset represents the calculated BMP9 EC_50_ for CTL and MUT ECFCs. **d** RT-qPCR quantification of I*D1* mRNA expression normalized to HPRT level in 4 CTL and 4 MUT (MUT-H1–H4) ECFCs following an 18 h stimulation with 10 ng/mL BMP9. Data are mean ± SEM of three independent stimulations presented as ΔΔCT compared to CTL NS. **e** Flow cytometric analysis comparing cell-surface ALK1 levels in 3 CTL vs 5 MUT ECFCs either carrying an *ALK1* missense mutations (MUT-H2, MUT-H4 and MUT-H6) or a nonsense mutations (MUT-H1 and MUT-H3). Isotypic control is illustrated in grey. One representative flow cytometry histogram of 3 is shown. **f** Quantification of the ALK1 cell-surface expression by flow cytometry (in percentage of in MUT ECFCs with missense or nonsense mutations compared to CTL ECFCs). Data are means ± SEM of three independent experiments. Two-way Anova followed by Sidak’s multiple comparisons tests were used for statistical analysis of **b**–**d**, except for the inset of **c** where Mann–Whitney test was used. Kruskal–Wallis test followed by Dunn’s multiple comparison’s test was used for **f**. For all panels, *ns* non-significant, *P < 0.05, **P < 0.01, ***P < 0.001 and ****P < 0.0001

To understand this surprising result, we next investigated the status of Smad1/5 phosphorylation induced by BMP9 in *ALK1*-mutated (*n* = 6) vs CTL ECFCs (*n* = 4). This analysis included the previously described ECFC clones that were studied by RNA-seq, as well as subsequently isolated clones from four additional newborns with different *ALK1* mutations and one additional CTL (Table 1; Fig. S4). We focused on BMP9 regulation only, as BMP9 and BMP10 showed similar transcriptomic responses (Fig. 1c). *ALK1* MUT ECFCs stimulated with BMP9 (10 ng/mL) for 1 h displayed similar levels of nuclear p-Smad1/5 immunofluorescence intensities to stimulated CTLs (Fig. 3a, b). To validate this result, we performed a BRE luciferase reporter assay in CTL (*n* = 4) and MUT (*n* = 6) ECFCs (Table 1; Figs. 1a, S4) stimulated with increasing doses of BMP9 (0.2–10 ng/mL) for 6 h. Both CTL and MUT ECFCs exhibited a clear dose-dependent response to BMP9 (Fig. 3c); yet, no significant differences were observed between the two groups (Fig. 3c), which displayed identical half maximal effective concentrations (EC_50_: 376 pg/mL for CTL vs 404 pg/mL for MUT ECFCs; inset Fig. 3c). We also quantified the mRNA levels of *ID1*, a target gene known to be strongly induced by BMP9, and found no difference between CTL (*n* = 4) and MUT (*n* = 4) ECFCs (Table 1; Fig. 3d). To examine whether the similar Smad1/5 activation in MUT vs CTL ECFCs was due to a compensation in ALK1 protein levels in *ALK1*-mutated ECFCs, we assessed the levels of membranous ALK1 by flow cytometry analysis in five different MUT ECFCs (Table 1) in comparison to 3 CTL ECFCs. We found that *ALK1*-mutated ECFCs carrying a missense mutation had a slightly lower level of membranous ALK1 level than CTL ECFCs, and those carrying an *ALK1* nonsense mutation displayed a 50% reduction in the level of cell surface ALK1 (Fig. 3e, f). Thus the similar Smad signaling response in CTL and *ALK1*-mutated ECFCs cannot be attributed to an elevated compensatory level of ALK1 in mutated ECFCs. Altogether, these results support that *ALK1*-mutated ECFCs maintain intact activation of the canonical Smad1/5 signaling pathway in response to BMP9 despite their reduced cell surface ALK1 levels.

### PAH patient-derived lung ECs carrying *ALK1* mutations display different transcriptomic profiles compared to CTLs at the basal state

**Fig. 4:**
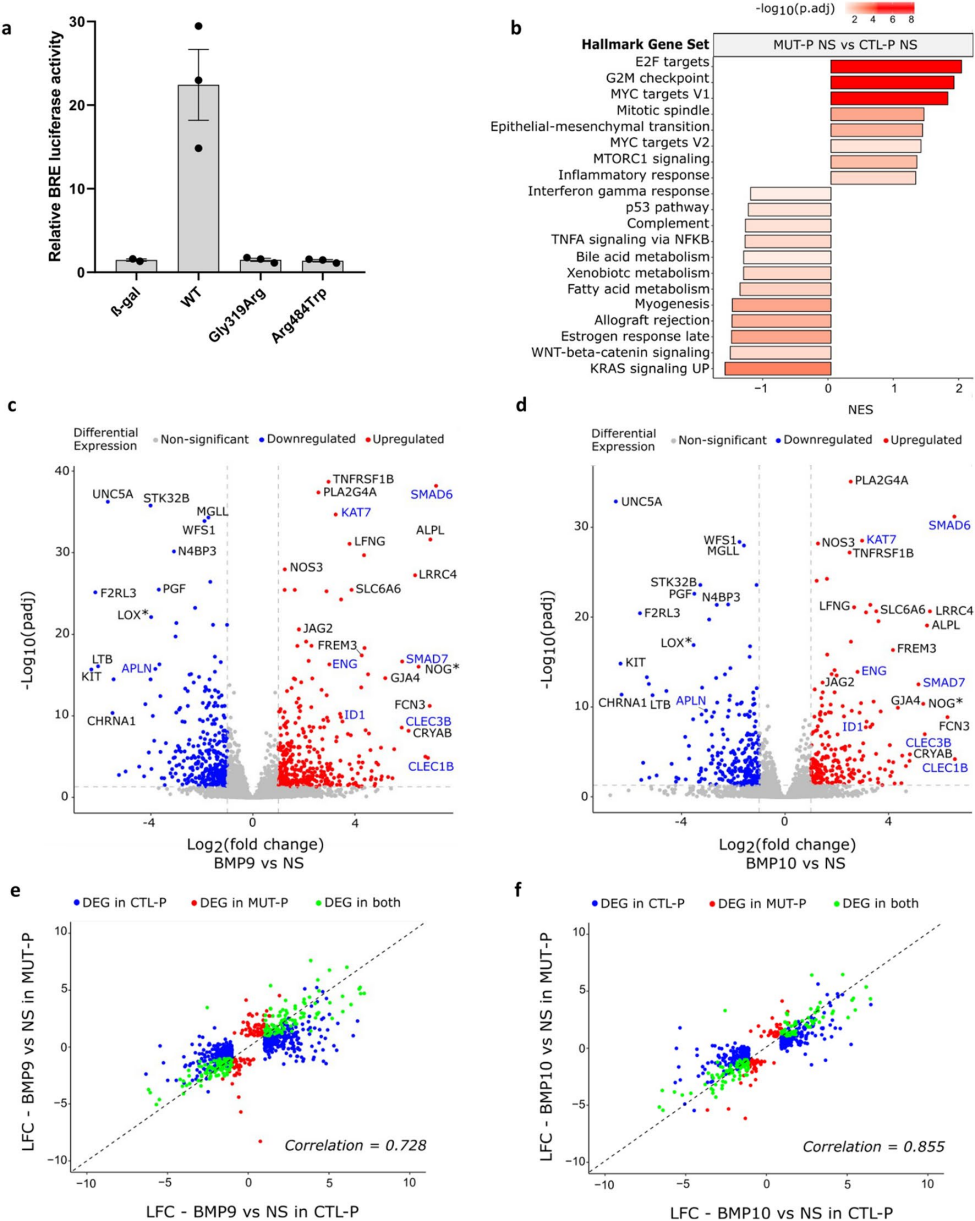
*ALK1*-mutated HMVECs display substantially different transcriptomic profiles compared to controls. **a** Relative BMP Response Element (BRE) luciferase activity measured in NIH-3T3 cells overexpressing either WT or mutant *ALK1* plasmids identified in *ALK1*-mutated HMVECs (p.Gly319Arg, MUT-P1) and (p.Arg484Trp, MUT-P2) that are included in the HMVEC RNA-seq analysis. BRE luciferase activities were normalized to renilla luciferase activities. Data shown are mean ± SEM from of three independent experiments. **b**–**f** 3 CTL and 2 *ALK1*-mutated (MUT-P1 and MUT-P2) HMVECs were stimulated or not with BMP9 or BMP10 (10 ng/mL) for 18 h. The experiment was repeated three times after which bulk RNA-seq analysis was performed. **b** GSEA performed using hallmark gene sets. The bar plot represents the top significant gene set categories enriched in non-stimulated MUT HMVECs compared to nonstimulated (NS) CTL HMVECs. Each bar represents a hallmark gene set and bars are ordered from top to bottom by decreasing order of enrichment score (NES). **c**, **d** Volcano plot representations showing global changes in gene expression in CTL HMVECs after BMP9 (**c**) or BMP10 (**d**) stimulation. DEGs with high LFC and high statistical significance are annotated. DEGs annotated in blue correspond to DEGs identified in CTL ECFCs (Fig. 1d, e). **e**, **f** Scatter plots comparing log_2_ fold change (LFCs) of DEGs regulated by BMP9 (**e**) or BMP10 (**f**) in CTL HMVECs vs MUT HMVECs. Pearson correlation is reported

Having not detected any differences in the transcriptomic nor in the early p-Smad1/5 response to BMP9 or BMP10 in newborn-derived *ALK1*-mutated ECFCs, we investigated the transcriptomic response in HMVECs carrying *ALK1*-mutations derived from transplanted sick lungs of two PAH patients (MUT-P1 and -P2, Table 3). The two *ALK1* mutations were missense mutations mapping to the kinase domain of ALK1 and were both confirmed as LOF mutations using the BRE luciferase assay (Fig. 4a).

**Table 3:** PAH patient characteristics

ID	Affected gene	Mutation		Type of mutation	Domain	Age	Gender	PAPm (mmHg)	NYHA Functional class	Therapies/diagnosis (for CTL)
MUT-P1	*ACVRL1*	c.955G>C	p.Gly319Arg	Missense	Kinase	14	Female	90	IV	Bosentan, sildenafil, treprostinil
MUT-P2	*ACVRL1*	c.1450C>T	p.Arg484Trp	Missense	Kinase	19	Female	100	II	Bosentan, sildenafil, epoprostenol
MUT-P3	*BMPR2*	del exon 11-13		Large deletion	Kinase + cytoplasmic tail	37	Female	74	IV	Bosentan, sildenafil, treprostinil
MUT-P4	*BMPR2*	c.314+3A>T		Splice site	Extracellular	14	Female		II	Bosentan
MUT-P5	*BMPR2*	c.901T>C	p.Ser301Pro	Missense	Kinase	26	Female	99	IV	None, transplanted immediately upon diagnosis
CTL-P1	–	–	–	–	–	80	Male	–	–	*Carcinoma*
CTL-P2	–	–	–	–	–	57	Female	–	–	*Invasive carcinoma*
CTL-P3	–	–	–	–	–	68	Male	–	–	*Epidermoid carcinoma*

List of *ALK1*- and *BMPR2*-mutated HMVECs that were isolated from explanted lungs of PAH patients, indicating the carried mutation, the type of mutation and the mutated or deleted *ALK1* or *BMPR2* domain, in addition to some characteristics and treatments of the patient. Control lung specimens were obtained from normal tissue collected at a distance from tumors from patients with localized lung cancer. The age, gender and diagnosis of the individuals were specified

*PAPm* mean pulmonary arterial pressure, *NYHA* New York Heart Association

To delineate the transcriptomic signature of these cells, RNA-sequencing was performed, using the same experimental conditions described for ECFCs, i.e. using 3 CTLs and 2 *ALK1*-mutated HMVECs from PAH patients (MUT-P1 and -P2, Table 3) that were either stimulated overnight with BMP9 or BMP10 (10 ng/mL) or were left nonstimulated (NS). Hierarchical clustering identified four separate clusters: (1) NS and stimulated MUT-P2 samples, (2) NS and stimulated MUT-P1 samples, (3) NS CTLs and (4) BMP9 and BMP10-stimulated CTL samples (from left to right, Fig. S5). This clustering showed that, unlike MUT-H ECFCs, the transcriptomes of MUT-P HMVECs were clearly different from those of CTLs. Interestingly, MUT-P HMVECs from each patient were separated into two different clusters (Fig. S5), highlighting variability between the two patients, which might be related to different disease stage or different treatments. The differential expression analysis (lLFCl ≥ 1 and *p*adj ≤ 0.05, Benjamini–Hochberg correction; Supplementary information SI3) comparing NS *ALK1*-mutated and NS CTL HMVECs identified 1261 protein-coding DEGs (Table 2), with both down-regulated (58%) and up-regulated (42%) genes. GSEA using hallmark gene sets in NS CTL vs* ALK1*-mutated HMVECs (Supplementary information SI4) revealed a positive enrichment of several gene sets involved in cell cycle (E2F targets, G2M checkpoint, MYC-targets and mitotic spindle) and a negative enrichment in genes related to various signaling pathways (KRAS signaling, WNT-beta-catenin signaling and TNFα signaling; Fig. 4b). Together, these data show that *ALK1*-mutated HMVECs derived from diseased lungs have a severely altered basal transcriptome compared to CTL HMVECs.

### BMP9 and BMP10 induce cell type-specific transcriptomic reponses

We then analyzed the BMP9 and BMP10 transcriptomic response in CTL HMVECs. Differential expression analysis identified 704 and 481 DEGs in BMP9- and BMP10-stimulated CTL HMVECs, respectively (Table 2). Although the number of DEGs was lower in BMP10 stimulated cells, the gene regulation patterns in BMP9- vs BMP10-stimulated CTL HMVECs were highly correlated (Pearson correlation coefficient = 0.844), further supporting, as for ECFCs (Fig. 1c), that BMP9 and BMP10 induce very similar transcriptomic responses in vitro under these experimental conditions. This similarity was also reflected in the volcano plots of BMP9- and BMP10-stimulated vs NS cells, which highlighted similar top dysregulated genes in terms of LFC or padj (Fig. 4c, d). Interestingly, by comparing BMP9/10-regulated genes in CTL HMVECs to those in CTL ECFCs, with the limitation that these data were derived from two independent RNA-sequencing, only a quarter of the DEGs was shared between ECFCs and HMVECs (27% for BMP9 and 26% for BMP10; Fig. S6a, b). *ID1*, *SMAD6*, *SMAD7*, *ENG*, *KAT7*, *CLEC1B*, *CLEC3B*, and *APLN* were identified as top targets in both ECFCs (Fig. 1d, e) and HMVECs (highlighted in blue in Fig. 4c, d). On the other hand, some top target genes were specific to HMVECs (e.g. *LOX* and *NOG*) (highlighted by an asterisk in Fig. 4c, d).

### *ALK1*-mutated HMVECs display slightly different transcriptomic responses to BMP9 and BMP10 compared to CTL HMVECs

We next analyzed the BMP9 or BMP10 response in *ALK1-*mutated vs CTL HMVECs. BMP9 and BMP10 stimulation resulted in the regulation of 295 and 206 DEGs, respectively (Table 2). This corresponds to around 60% less DEGs compared to stimulated CTLs. This decrease in the number of DEGs suggests that *ALK1*-mutated HMVECs might possess a reduced capacity to respond to BMP9 or BMP10. When comparing the LFCs of each list of dysregulated genes in stimulated CTL vs MUT HMVECs, we obtained fairly high Pearson correlation coefficients (0.728 for BMP9 and 0.855 for BMP10; Fig. 4e, f). However, these correlations were lower than the ones obtained in ECFCs (Fig. 2a, b), suggesting that the BMP9 and BMP10 responses in *ALK1*-mutated HMVECs might be more affected than in *ALK1*-mutated ECFCs. Since we are simultaneously analyzing two variables (genotype and stimulation), and because there was a clear difference between CTL and MUT HMVECs at the basal level (Table 2), we performed a two-factor analysis as previously described [26] (See Materials and Methods section in Supplementary information) considering the two factors: genotype and BMP9 or BMP10 treatment (Supplementary information SI5). We found 44 protein-coding interaction term genes differentially regulated by BMP9 in CTL versus *ALK1*-mutated HMVECs and 15 in response to BMP10, which were all shared with BMP9 stimulation (marked by an asterisk, Benjamini–Hochberg adjusted for multiple comparisons, *p*adj < 0.05; Fig. S7a). On the other hand, when applying the same kind of analysis on CTL and MUT ECFCs, we could not detect any interaction term genes, in accordance with the highly similar transcriptomic responses to BMP9/BMP10 between MUT and CTL ECFCs. Then, we hypothesized that this difference between MUT HMVECs and MUT ECFCs could be due to a further reduction in ALK1 level in MUT HMVECs below a critical threshold. Nevertheless, flow cytometric analysis revealed similar cell-surface ALK1 levels in MUT HMVECs compared to their control counterparts (Fig. S8).

Out of the 44 interaction term genes identified in MUT HMVECs, we selected 25 genes (highlighted in blue in Fig. S7a) for validation by RT-qPCR on independent BMP9 stimulations. The selection was based on genes showing only minimal interindividual heterogeneity between members of each group (CTL or MUT group; data not shown). The regulation trend of each of the 25 selected genes was confirmed by RT-qPCR (Fig. S7b), validating the robustness of the two-factor analysis.

### *LFNG* (lunatic fringe) shows impaired regulation by BMP9 in *ALK1*-mutated HMVECs, ECFCs and HUVECs

**Fig. 5:**
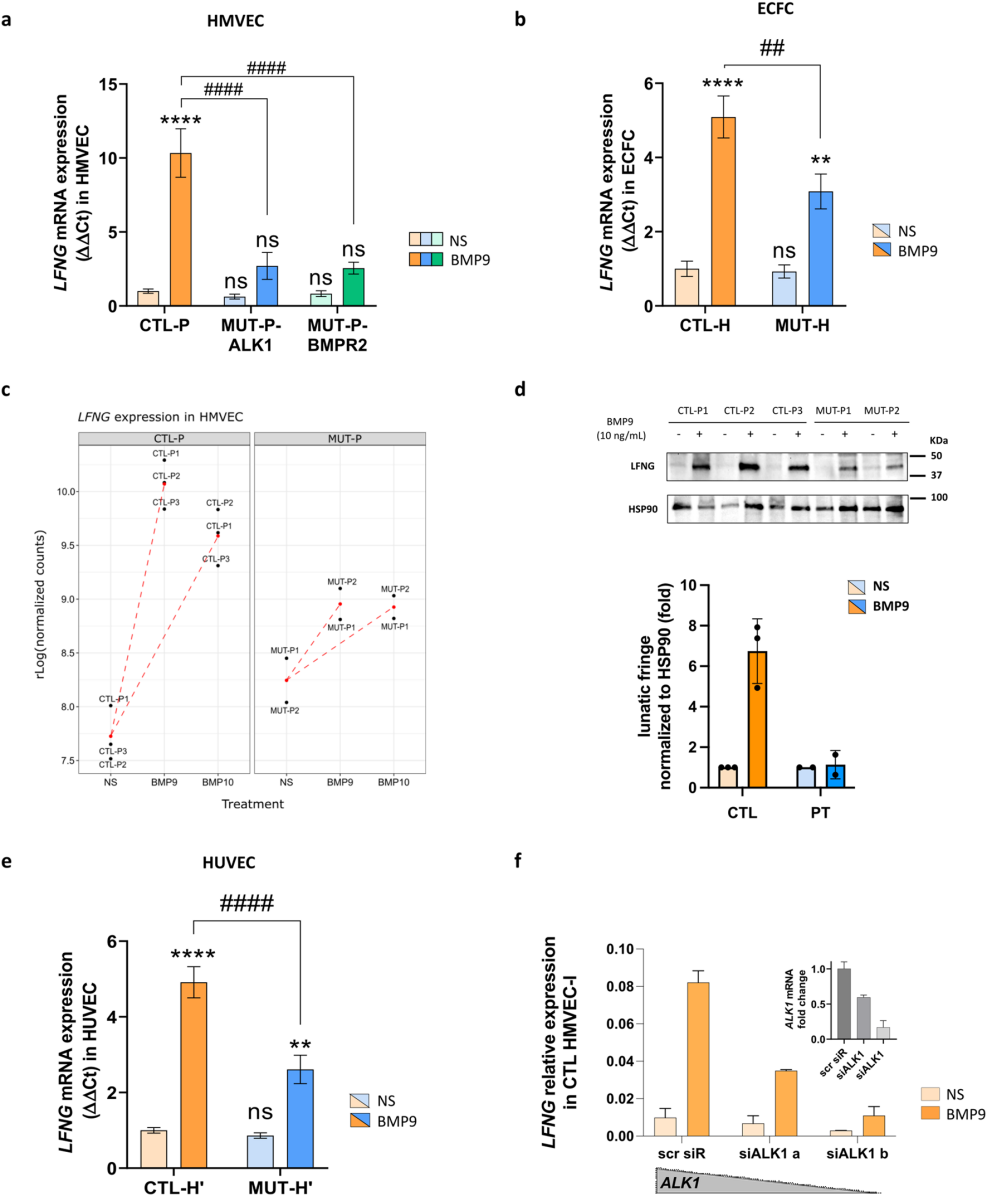
Regulation of *LFNG* mRNA expression by BMP9 in CTL and *ALK1*-mutated HMVECs, ECFCs and HUVECs. **a**, **b** RT-qPCR quantification of the mRNA expression level of *LFNG* in 3 CTL, 2 *ALK1*-mutated (MUT-P1 and -P2) and 3 *BMPR2*-mutated HMVECs (MUT-P3–P5) (**a**), 3 CTL and 4 *ALK1*-mutated ECFCs (MUT-H1–H4) (**b**). *LFNG* mRNA expression level was normalized to *HPRT* mRNA expression and presented as ΔΔ*Ct* compared to mean CTL NS. Data shown are mean ± SEM of at least three independent stimulations. **c** Count plot representation showing the regularized log transformed counts of *LFNG* mRNA in CTL-P and *ALK1*-MUT-P HMVECs in nonstimulated (NS) and BMP9 or BMP10 stimulated cells. **d** Western blot analysis of 3 CTL (1–3) and 2 A*LK1*-mutated HMVECs (MUT-P1–2) that were either NS or stimulated for 24h with 10 ng/mL BMP9. Cell lysates were resolved by 4–20% SDS–PAGE and immunoblotted with antibodies against lunatic fringe or against HSP90 (loading control). The resulting blots are shown along with quantification of the mean lunatic fringe signal normalized to HSP90. **e** RT-qPCR quantification of the mRNA expression level of *LFNG* in 3 CTL (CTL-H′1–3) and 3 *ALK1*-mutated (MUT-H′3–5) HUVECs. *LFNG* mRNA expression level is normalized to *HPRT* mRNA expression and presented as ΔΔCT compared to mean CTL NS. Data shown are mean ± SEM of at least three independent stimulations. **a**, **b**, **e**, Two-way Anova followed by Sidak’s multiple comparisons test were used for statistical analysis of panels. *ns* non-significant, **P < 0.01 and ****P < 0.0001 vs NS and ^##^*P* < 0.01 and ^####^*P* < 0.0001 vs CTL. **f** 2 CTL HMVECs were treated either with scrambled siRNA (siScr) or two different concentrations of siRNA against *ALK1* (siALK1 a and b) to generate a gradient of ALK1 expression and then stimulated with 10 ng/mL BMP9 for 18 h. *LFNG* mRNA expression normalized to *HPRT* mRNA level is presented as 2^−Δ*Ct*^. Data shown are mean ± SD of 2 CTL HMVECs. Inset represents *ALK1* mRNA expression presented as ΔΔ*Ct* compared to scrambled siRNA-transfected cells

Among the 25 genes validated, we focused on 6 (*LFNG*, *JAG2*, *TNFRSF1B*, *SLC6A6*, *SOX13* and *CEBPG*) that were also identified as DEGs in response to BMP9 and BMP10 in CTL ECFCs with the same sense of regulation, making them good candidates to compare the effect of *ALK1* mutation on gene regulation by BMP9 across the different EC models. The BMP9 regulation of all 6 genes was confirmed by RT-qPCR in CTL HMVECs (Figs. 5a, S9a), and consistent with the two-factor analysis, their induction was repressed in *ALK1*-mutated HMVECs, except for *SLC6A6*, whose difference between CTL and MUT HMVECs did not reach statistical significance (Fig. S9a). Due to the limited number of accessible PAH donors with *ALK1* mutations, and to test whether LOF mutations in another component of the receptor complex would affect the response similarly, we tested *BMPR2*-mutated HMVECs derived from transplanted lungs of 3 PAH patients (Table 3), and found comparable regulation patterns to *ALK1*-mutated HMVECs for all 6 genes (Figs. 5a, S9a).

Then, we tested the BMP9 regulation of these genes in CTL and *ALK1*-mutated ECFCs (*n* = 4) using RT-qPCR. BMP9 stimulation upregulated the mRNA expression levels of all six genes in CTL ECFCs (Figs. 5b, S9b). In accordance with the results of the two-factor analysis in ECFCs, the regulation of the aforementioned genes in *ALK1*-mutated cells showed either similar or slightly weaker regulation by BMP9 compared to CTLs (Fig. S9b), with the exception of *LFNG* (Fig. 5b). In HMVECs, using RT-qPCR, *LFNG* mRNA was strongly upregulated by BMP9 in CTLs (10.3 folds), but this upregulation was strongly reduced in *ALK1*-mutated (2.7 folds) and *BMPR2*-mutated HMVECs (2.6 folds; Fig. 5a), mirroring the result of the two-factor analysis generated from the RNA-seq data in these cells, as illustrated in the count-plot representation (Fig. 5c). The dysregulation of *LFNG* was also validated at the protein level following a 24 h stimulation of 10 ng/mL BMP9 in CTL and *ALK1*-mutated HMVECs (6.49 folds in CTL vs 1.17 folds in *ALK1*-mutated HMVECs; Fig. 5d). In ECFCs, while CTL cells displayed a significant upregulation of *LFNG* expression by BMP9 (5.1 folds), *ALK1*-mutated ECFCs presented a significantly weakened upregulation of *LFNG* (3.1 folds, *n* = 4; Fig. 5b). Together, these results support the hypothesis that *LFNG* transcriptional regulation by BMP9 could be affected by ALK1 heterozygosity, but did not allow to conclude on the other interaction term genes studied.

To further test this hypothesis, we studied the BMP9 response in human umbilical vein endothelial cells (HUVECs), another type of ECs that can be isolated from newborns. RT-qPCR for *LFNG*, *JAG2*, *TNFRSF1B*, *SLC6A6*, *SOX13* and *CEBPG* were performed on 3 CTLs and 3 LOF *ALK1*-mutated HUVECs (CTL-H and MUT-H) stimulated or not with BMP9 for 18 h. These genes were all induced in response to BMP9 in CTL HUVECs, although SOX13 stimulation did not reach significance (Figs. 5e, S9c). As for HMVECs and ECFCs (Fig. 5a, b), we found a significant decrease in the level of induction of *LFNG* mRNA expression by BMP9 in *ALK1*-mutated HUVECs compared to CTLs (FC = 4.9 in CTL-H′ and 2.6 in MUT-H′; Fig. 5e). The BMP9 regulation of *JAG2*, *TNFRSF1B* and *SLC6A6* mRNA expressions were also found to be significantly reduced in *ALK1*-mutated vs CTL HUVECs (Fig. S9c).

To directly relate the BMP9-induced upregulation of *LFNG* to functional ALK1 levels, we tested *LFNG* induction in CTL HMVECs whose ALK1 expression level was reduced using an siRNA approach. Reducing *ALK1* mRNA levels in CTL HMVECs by 50 or 90% (siALK1 a and siALK1 b, respectively; inset in Fig. 5f) suppressed *LFNG* induction by BMP9 in a dose-dependent manner (Fig. 5f).

Together, these data show that *ALK1* heterozygosity could impair the BMP9 regulation of the mRNA expression of *LFNG* and possibly other genes, depending on the endothelial cellular model.

## Discussion

To our knowledge, this is the first study assessing the BMP9 and BMP10 transcriptomic responses in ECs carrying heterozygous *ALK1* mutations. In this work, we performed RNA-seq analyses on two types of primary *ALK1*-mutated cells: (1) ECFCs derived from newborns with an HHT-affected parent and (2) HMVECs derived from severely ill explanted lungs of end-stage PAH patients.

The first interesting point from this study was the comparison between BMP9 and BMP10 transcriptomic responses in ECs. BMP9 and BMP10 have been identified as two high affinity ligands for ALK1 with very similar affinities. BMP9, but not BMP10, can also bind the type I receptor ALK2, yet with a much lower affinity than ALK1 [27]. In addition, different affinities for the type II receptors to BMP9 vs BMP10 have been described [28]. Here, we show that BMP9 and BMP10, despite the reported differences in receptor binding affinities, induced highly similar transcriptomic responses in both ECFCs and HMVECs. This is in accordance with recent work that compared the transcriptomic regulation by precursor forms of BMP9 and BMP10 at lower concentrations in pulmonary arterial ECs [29]. Nonetheless, these in vitro findings do not rule out specific in vivo roles that can arise from differences in spatiotemporal expression patterns, localization within the extracellular matrix or availibility in the circulation as previously discussed [30].

Another interesting point was the use of two different EC types (ECFCs and HMVECs) for studying BMP9 and BMP10 transcriptomic responses. With the limitation that these two RNA-seq analyses were performed independently, it is interesting to note that the transcriptomic profiles of ECFCs and HMVECs in response to BMP9 or BMP10 stimulation were not largely overlapping, with only 26–27% of genes commonly regulated in both cell types (Fig. S6a, b). Nonetheless, BMP9 or BMP10-induced gene regulation patterns between ECFCs and HMVECs were still fairly correlated, with Pearson correlation coefficients reaching 0.71 and 0.75 for BMP9 and BMP10, respectively. This suggests that a proportion of the DEGs identified in one EC type are not detected in the other because they don’t reach the set thresholds rather than displaying differential patterns of gene regulation. On the other hand, the other group of genes presenting distinct regulation patterns between the two cell types indicate that the studied cells retain organotypic specificities despite being cultured in vitro. This specificity could be attributed to predetermined BMP Smad binding sites in specific EC types as already described [31]. Among the cell type-specific targets, we identified *LOX* as a strongly downregulated target in response to BMP9 or BMP10 in HMVECs but not in ECFCs. Interestingly, *LOX*, which codes for a lysyl oxidase that is implicated in crosslinking of extracellular matrix components, was previously found to be elevated in the proliferating pulmonary endothelium of PAH patients [32]. We also detected *NOG*, encoding the strong BMP antagonist noggin [33, 34] as a target upregulated by BMP9/10 in HMVECs, but not in ECFCs.

This work also unexpectedly revealed the high similarity in the BMP9 or BMP10 response between CTL and *ALK1*-mutated ECFCs, both at the transcriptomic level and at the Smad1/5 activation level. This result is rather surprising, as it demonstrates, for the first time, that losing one functional *ALK1* allele does not considerably affect the downstream Smad signaling response. The intact activation of the pathway in mutated ECFCs is not likely attributable to the high dose of BMP9 and BMP10 used in this study, as similar results were obtained using low doses of BMP9 in the BRE luciferase assay on CTL and MUT ECFCs (Fig. 3c). Nevertheless, complementary transcriptomic studies using lower BMP9 or BMP10 doses would be necessary to conclusively eliminate this possibility. The intact activation cannot be explained by a compensation in ALK1 expression either, as decreased levels of membranous ALK1 were detected in the heterozygote ECFCs (Fig. 3f). Our findings thus support the hypothesis that 50% WT ALK1 is sufficient for driving normal canonical signal transduction.

More importantly, the nearly identical transcriptomic responses to BMP9 or BMP10 between CTL and *ALK1-*mutated ECFCs suggests that haploinsufficiency might not be the cause of HHT development. This result is in accordance with the recent identification of a bi-allelic loss of *ALK1* or *ENG* in at least 50% of tested cutaneous telangiectasia samples isolated from HHT patients [35], supporting the hypothesis that a second ‘knudsonian’ somatic hit in the other allele is necessary to drive HHT pathogenesis. This notion is not novel to the vascular anomalies field, as it was already shown for venous, glomuvenous and cerebral cavernous malformations [36, 37]. These somatic mutations could explain why some lesions develop only focally in HHT patients, and how related patients, carrying the same mutation, can develop different manifestations of the disease. However, this hypothesis has not yet been validated neither in liver or lung AVMs or in diffuse lesions.

In contrast to *ALK1*-mutated ECFCs isolated from newborns, nonstimulated *ALK1*-mutated HMVECs isolated from the lungs of endstage PAH patients revealed strong dysregulations in gene expression compared to controls at the basal level (1261 DEGs, Table 2). This might not be surprising, as these cells have been exposed to a pathogenic, likely inflammatory environment as is usually described for lungs of PAH patients [38] and as evidenced by the enrichment in proliferation and inflammation-related gene sets in the mutated cells by our GSEA (Fig. 4b). Due to these large transcriptomic differences already at the basal level, it was difficult to pinpoint which genes are differently regulated by BMP9 and BMP10 in the presence of an *ALK1* mutation, regardless of their initial baseline differences. Hence, using a two-factor analysis, which takes into account the two variables (genotype and BMP stimulation), we identified and then validated by RT-qPCR 25 such genes in *ALK1*-mutated HMVECs and discovered that *LFNG* was significantly dysregulated in the three tested *ALK1*-mutated EC types (HMVECs, ECFCs and HUVECs), when assessed by RT-qPCR. Intriguingly, the BMP9 regulation of *LFNG* was impaired in *ALK1*-mutated ECFCs, despite their normal Smad1/5 activation. One possible explanation for that, as proposed by Morikawa et al. [31], is that different Smad1/5 binding sites have different affinities for Smad1/5 complex, with some high affinity sites, such as that of *ID1*, and other low affinity ones, such as those of *JAG1* and *HEY1*, thus requiring higher or more sustained levels of Smad1/5 activation or the involvement of other DNA binding protein partners for their regulation [31]. In that same study, *LFNG*’s promoter was shown by chromatin immunoprecipitation-sequencing to be bound by Smad1/5 in response to BMP9 stimulation in HUVECs [31]. *LFNG* could be one of these genes with low Smad1/5 binding affinity and/or needing other Smad binding partners, making it more sensitive to ALK1 heterozygosity. Interestingly, by revisiting recently published transcriptomic data of two mouse HHT models (*Alk1* deleted in liver endothelial cells [39] and *Smad4* deleted in endothelial cells [40]), we found significantly reduced expression of *Lfng* compared to WT mice. *ALK1*-mutated HUVECs, but not *ALK1*-mutated ECFCs, additionally displayed significant dysregulations in *JAG2*, *TNFRSF1B* and *SLC6A6* mRNA expression, suggesting that HUVECs might be more sensitive to *ALK1* LOF than ECFCs. This could be due to the fact that HUVECs are derived from a vessel environment in contrast to ECFCs, which are circulating cells.

Interestingly, *LFNG* and *JAG2* are two components of the Notch signaling pathway, which is a master regulator of tip/stalk cell differentiation and arterial specification [41]. Furthermore, multiple Notch-defective mouse models were reported to develop AVMs [42–45], which represent a major pathological feature of HHT. In addition, BMP and Notch pathways synergistically upregulate several shared transcription factors such as Hey1, Hey2 and Hes1 [46, 47]. Here, we shed light on a new intersection point between BMP and Notch axes, through BMP9/10-mediated regulation of *LFNG*. This gene codes for lunatic fringe, a glycosyl transferase that post-translationally modifies Notch1, leading to inhibition of its activation by Jagged ligands while enhancing its activation by Delta-like ligands (Dll) [48–50]. It is noteworthy that reduced activation of Dll4-mediated Notch signaling results in excessive sprouting during the activation phase of angiogenesis [51] and promotes cell cycle reentry during the maturation phase [52]. Knowing that *Alk1*-depleted HHT mouse models display hypersprouting and increased vascular density in their developing retinas [53], it is plausible that suppressed upregulation of *LFNG* by BMP9 in the presence of *ALK1*-mutations might be contributing to this phenotype by mitigating Dll4 activation. Interestingly, during the submission of this work, a preprint by Ristori et al. that also highlighted a crosstalk between BMP9 and *LFNG* in regulating Notch signaling was deposited in BioRxiv [54].

All in all, this work provides, to our knowledge, the first in vitro line of evidence that *ALK1* heterozygosity on its own does not drastically impair the response of ECs to BMP9 nor BMP10, neither at an early step of signal transduction (phosphorylation of Smad1/5), nor downstream at the transcriptomic level, supporting that one functional receptor could be enough at least for canonical signaling. We also show that the two high affinity ALK1 ligands, BMP9 and BMP10, induce highly similar transcriptomic changes in vitro, pointing to an overlapping function in this context. Interestingly, through deeper investigations, we could identify at least one gene, i.e. *LFNG*, whose regulation by BMP9 was weakly impaired in newborn heterozygous *ALK1*-mutated ECs, but more intensely suppressed in *ALK1* or *BMPR2*-mutated HMVECs from PAH patients. Altogether, our findings suggest that heterozygous *ALK1* mutations could be priming events awaiting further triggers for driving lesion development. One limitation of this study is that it was conducted under static conditions, while ECs are typically exposed to physiological flow, which has been demonstrated to influence BMP9/BMP10-ALK1-endoglin signaling [55]. It will thus be interesting to study how these *ALK1*-mutated cells behave under shear stress or in response to angiogenic or inflammatory triggers. In parallel, future studies investigating the role of *LFNG*, and more largely the Notch signaling pathway, are needed to establish their implication in driving early HHT pathogenesis.

### Supplementary Information

Below is the link to the electronic supplementary material.

Supplementary file1 (DOCX 31 kb)

Supplementary file2 (TIFF 2152 kb)

Supplementary file3 (TIFF 2152 kb)

Supplementary file4 (TIFF 2152 kb)

Supplementary file5 (TIFF 2152 kb)

Supplementary file6 (TIFF 2152 kb)

Supplementary file7 (TIFF 2152 kb)

Supplementary file8 (TIFF 2152 kb)

Supplementary file9 (TIFF 2197 kb)

Supplementary file10 (TIFF 2152 kb)

Supplementary file11 (XLSX 59956 kb)

Supplementary file12 (XLSX 44991 kb)

Supplementary file13 (XLSX 25 kb)

Supplementary file14 (XLSX 33358 kb)

Supplementary file15 (XLSX 18 kb)

Supplementary file16 (XLSX 12 kb)

Supplementary file17 (DOCX 13 kb)

Supplementary file18 (DOCX 15 kb)
