# Supplementary material for: Impact of heterozygous ALK1 mutations on the transcriptomic response to BMP9 and BMP10 in endothelial cells from hereditary hemorrhagic telangiectasia and pulmonary arterial hypertension donors
Source: Angiogenesis. 2024 Jan 31;27(2):211–27. doi: 10.1007/s10456-023-09902-8 (PMC11021321; doi:10.1007/s10456-023-09902-8)
Supplement: Supplementary file 1 — Supplementary file1 (DOCX 31 kb) [file 10456_2023_9902_MOESM1_ESM.docx]

Supplementary information

*Materials and Methods*

The study was approved by the local research ethics committee (Hospice civils de Lyon, CPP 2021-A01792-39) and by the French Medical Products Agency (ANSM). Written informed consent was obtained from all patients in accordance with national regulations. The trial was conducted in accordance with the principles of the Declaration of Helsinki[1] and Good Clinical Practice guidelines. This trial was registered with the ClinicalTrials.gov Identifier #NCT05632484.

(<https://clinicaltrials.gov/ct2/show/NCT05632484?cond=HHT&map_cntry=FR&draw=3&rank=12>).

*Primary endothelial cell isolation*

Human umbilical cord blood (UCB) samples (35–110 mL) were collected in heparin-coated syringes from 6 newborns carrying HHT-linked *ALK1* mutations and 4 healthy subjects (Table 1). Isolation of endothelial colony-forming cells (ECFCs) was performed as recommended by the Vascular Biology Standardization Subcommittee[2]. UCB samples were diluted at a 1:3 ratio in RPMI 1640 medium (Gibco) supplemented with 2% fetal bovine serum (FBS; Biosera). Then, mononuclear cell (MNC) fractions were isolated through density gradient centrifugation using 1.077 g/ml Pancoll solution (Pan Biotech) followed by successive washing steps. Finally, MNC were plated in 10μg/mL fibronectin-coated 24-well tissue culture plates in microvascular endothelial cell growth medium-2 (EGM-2 MV; Lonza) with 10% FBS at a density of 5 × 10^6^ cells/cm^2^. Cells were incubated in 5% CO_2_ at 37°C and the medium was changed daily during the first 7 days and every other day thereafter. Following the first passage, the serum composition was reduced to 5%. ECFCs were used up to 45 days after cord blood processing.

In parallel, human umbilical vein endothelial cells (HUVECs) were isolated, as previously described[3], from the umbilical vein of a number of newborns from which ECFCs were isolated (Table 1).

Human microvascular endothelial cells (HMVECs) were isolated from the explanted lungs of PAH patients during lung transplantation as previously described[4, 5] (Table 3). Control lung specimens were obtained from patients without evidence of pulmonary vascular disease who underwent lobectomy or pneumonectomy for localized lung cancer, with the normal tissue collected at a distance from the tumors.

*Cell culture*

ECFCs, HMVECs and HUVECs were maintained in EGM-2 MV medium (Lonza). For RNA-sequencing and RT-qPCR, cells were washed twice with phosphate buffered saline (PBS) and were incubated for 18h in EBM-2 (Endothelial Basal Medium, Lonza) with or without 10ng/ml recombinant human BMP9 (3209-BP, R&D Systems) or recombinant human BMP10 (2926-BP; R&D Systems). Murine NIH-3T3 fibroblasts were maintained in high glucose, sodium pyruvate and GlutaMAX-supplemented Dulbecco’s modified Eagle medium (DMEM; Gibco) with 10% FBS (Biosera) and 1% Pen/Strep (Gibco).

*Immunofluorescence*

For VE-cadherin staining, ECFCs were seeded at confluency in glass Lab-Tek Chamber Slides (Thermo Fisher Scientific), fixed the next day with 4% paraformaldehyde, permeabilized with 0.1% Triton X100 in PBS, saturated with 1% bovine serum albumin (BSA; Sigma-Aldrich) in PBS and incubated at 4°C for 1h with anti-VE-cadherin (1:100; #2158; Cell Signaling Technology). For detection, the cells were subsequently incubated with alexa fluor 488 donkey anti-rabbit IgG (AB_2340620, Jackson ImmunoResearch) for 30min at room temperature. Nuclei were counterstained with Hoechst 33342 (Sigma-Aldrich) and images were acquired using Axio Imager 2 (ZEISS) and analyzed on ZEN Microscopy Software.

Phospho-Smad1/5 (p-Smad1/5) immunostaining in ECFCs was performed as previously described[6]. 20,000 cells/well were seeded in 96-well black cell culture microplate (Greiner Bio-One 655090) for 24h.Then, cells were starved for 2hr in EBM-2 and stimulated for 1hr with 10ng/mL recombinant human BMP9 (R&D Systems). Cells were then fixed with 4% paraformaldehyde, permeabilized with 0.2% Triton X100 in PBS, saturated with 3% BSA in PBS and incubated overnight at 4°C with anti-p-Smad1/5 (1:800; #9516; Cell Signaling Technology). The cells were subsequently incubated with alexa fluor 488 donkey anti-rabbit IgG (AB_2340620, Jackson ImmunoResearch) and nuclei were counterstained with Hoechst 33342. Images were acquired using IN Cell Analyzer 2500HS widefield fluorescence microscope using a 20X objective. At least 16 different fields were imaged/well and 3 independent experiments were performed with 2 technical replicates each. To allow comparison between wells, the exposure time was kept constant across all wells. The acquired images were then analyzed using InCarta software (General Electrics Healthcare, USA).

*Flow cytometry*

Cells were seeded 25000 cells/cm^2^ and allowed to grow for 48 hours. Cells were detached using trypsin/EDTA solution (CC-5012; Lonza) diluted at 0.000714%, resuspended in 2% BSA in PBS at 1x10^7^ cells/mL and incubated with one of the following antibodies: fluorescein isothiocyanate (FITC)–conjugated mouse anti-human CD31 (clone WM59; 557508; BD Pharmingen), FITC-conjugated mouse anti-human CD45 (clone HI30; 560976; BD Pharmingen), phycoerythrin (PE)-conjugated mouse anti-human CD144 (clone 55-7H1; 560410; BD Pharmingen), PE mouse anti-human CD146 (clone P1H12; 550315; BD Pharmingen), unconjugated goat anti-human ALK1 antibody (AF370; R&D Systems) followed by alexa fluor 488 donkey anti-goat IgG (AB_2340430, Jackson ImmunoResearch) or an isotype control antibody. Ten to twenty thousand cells were analyzed on the BD FACSMelody sorter (BD Biosciences) using FCS Express Flow Cytometry Software.

*Site-directed mutagenesis*

Plasmids encoding the ALK1 mutations understudied were generated by polymerase chain reaction through site-directed mutagenesis of a pcDNA3-1(+) plasmid encoding WT N-terminal HA-tagged ALK1 using the QuikChange Lightning kit (Agilent) following the manufacturer’s instructions. All mutated plasmids were verified by full sequencing (Eurofins). Primer sequences used for mutagenesis are listed in Suppl Table 1.

*Luciferase reporter assay*

NIH-3T3 cells in white 96-well culture plates (Greiner) were transfected in Opti-MEM (Invitrogen) using lipofectamine 2000 (Invitrogen) with 75ng pGL3(BRE)2-luc, 30ng pRL-Tkluc and 5ng of plasmids encoding either HA-tagged WT or mutant ALK1 as previously described[7]. Five hours post transfection, cells were stimulated with or without recombinant human BMP9 (100 pg/mL) for 18 hours. For direct luciferase activity measurements in primary ECFCs, the cells in white 96-well culture plates (Greiner) were transfected in Opti-MEM using lipofectamine 3000 (Invitrogen) with 40ng pGL3(BRE)2-luc and 60ng pRL-Tkluc. Three hours post transfection, ECFCs were stimulated with or without recombinant BMP9 in increasing concentrations (0.2 ng/mL–10 ng/mL) for 6 hours. Firefly and renilla luciferase activities were sequentially measured with twinlite Firefly and Renilla Luciferase Reporter Gene Assay System (Perkin Elmer) using the SPARK multimode microplate reader (Tecan) and final luciferase activities were reported as firefly luciferase activities normalized to renilla luciferase activities.

*RNA sequencing and bioinformatic analysis*

Cells from each donor were seeded in 3 culture vessels and were stimulated or not overnight with 10ng/ml of BMP9 or BMP10. Stimulations were repeated two or three times for each donor, generating technical replicates. After an overnight stimulation, the cells were trypsinized, spun and frozen as dry pellets at -80°C prior sending to Genewiz (https://www.genewiz.com) for RNA extraction, quality control, library preparation and RNA-sequencing. Mapped reads and sample metadata were imported into R software (version 4.0.3[8]), and loaded in DESeq2 R package (version 1.30.1[9]). Replicates were collapsed using the “collapseReplicates” function from the DESeq2 package. In order to reduce noise, genes that had less than 150 counts across all samples were filtered out. Differential gene expression analysis was performed using the DESeq2 package, and the Wald test was used to determine the significance of log_2_ fold change. Pairwise comparisons between conditions, as well as intercept terms for two-factor analysis, were retrieved using the “results” function from the DESeq2 package. Regularized log transformed data was used to perform Principle Component Analysis (PCA) and to plot expression heatmaps and countplots. Heatmaps were generated using the “ComplexHeatmap” (version 2.6.2[10], R package, and hierarchical clustering was based on Euclidian distance and Complete linkage. Gene set enrichment analysis (GSEA)[11] was performed using the “clusterProfiler” R package (version 3.18.1[12]). The ordering vector for GSEA was based on the stat value of the Wald test. Gene set on which GSEA was based was “Hallmark pathways”, obtained from Broad Institutes MsigDB (version 7.5.1[13]). All p-values were adjusted for multiple testing using the Benjamini-Hochberg procedure, to obtain a false discovery rate (FDR). Only protein-coding DEGs are shown in volcano and scatter plots.

*RNA extraction and RT-qPCR*

Cells were lysed and RNA was extracted using the NucleoSpin RNA kit (Macherey-Nagel) according to the manufacturer’s instructions. 1µg RNA was reverse-transcribed using iScript cDNA Synthesis Kit (Bio-Rad) in a T100 thermal cycler (Bio-rad), and quantitative PCR was performed on 1/10 diluted cDNA samples using SsoAdvanced Universal SYBR Green Supermix (Bio-Rad) in a CFX96 Real-Time System, (Bio-Rad). Data analysis was performed with CFX Manager Software V3.1 (Bio-Rad) and relative expression levels were calculated using the delta Ct (ΔCt) method with *HPRT* serving as the housekeeping gene. Alternatively, fold changes in expression levels were calculated using the Livak’s ΔΔCt method. Primer sequences used for RT-qPCR are listed in Suppl Table2.

*Western blotting*

CTL and ALK1-mutated HMVECs seeded in 6-well culture plates were starved for 2hr in EBM2 followed by a 24hr stimulation with 10ng/mL BMP9. Then, cells were washed twice with ice cold phosphate-buffered saline and lysed in radioimmunoprecipitation assay buffer supplemented with P8340 protease inhibitor cocktail and phosphatase inhibitor cocktails 2 and 3 (Sigma-Aldrich). Proteins were extracted from the lysates then quantified using the microBCA protein Assay kit (ThermoFisher Scientific). 10µg of proteins from each sample were subjected to sodium dodecyl sulfate–polyacrylamide gel electrophoresis on 4-20% Precast Protein Gels (Bio-rad) followed by blotting onto nitrocellulose membranes using the Mini Trans-Blot system (Bio-rad). Following blocking with instant block buffer (Euromedex ) for 10mins, the membranes were incubated with anti-lunatic fringe (D6V2V; #66472; Cell Signaling Technology) or anti-HSP90 (C45G5; **#**4877; Cell Signaling Technology) as a loading control. Appropriate secondary antibodies were incubated prior to incubation with SuperSignal™ West Femto Maximum Sensitivity Substrate (Thermo Scientific) and images were captured using ChemiDoc Imaging System (Bio-Rad) and quanified with Image lab software V6.1 (Bio-Rad).

*RNA interference*

CTL HMVECs (250,000 cells/well in 6-well plates) were transfected using Lipofectamine RNAiMAX Transfection Reagent (2.5µL/well, Invitrogen) with either Silencer Negative Control #1 siRNA (AM4611, Ambion) at a final concentration of 1nM or Silencer Select pre-designed siRNA directed against human ALK1 (siALK1, 4392420, siRNA ID s987, Ambion) at a final concentration of 0.0035nM or 1nM, to induce different degrees of ALK1 silencing. Lipofectamine and siRNA mixes were incubated at room temperature for 20mins followed by cell transfection in Opti-MEM (11058021, Gibco). Five hours later, an equivalent volume to Opti-MEM of EGM-2MV medium with 10% FBS was added per well to obtain 5% final composition. Forty-eight hours post-transfection, cells were stimulated or not with BMP9 10ng/mL for 18hrs to study *LFNG* regulation by BMP9 as a function of ALK1 level.

*Supplementary References*

1. World Medical Association (2013) World Medical Association Declaration of Helsinki: ethical principles for medical research involving human subjects. JAMA 310:2191–2194. https://doi.org/10.1001/jama.2013.281053

2. Smadja DM, Melero‐Martin JM, Eikenboom J, et al (2019) Standardization of methods to quantify and culture endothelial colony‐forming cells derived from peripheral blood: Position paper from the International Society on Thrombosis and Haemostasis SSC. J Thromb Haemost 17:1190–1194. https://doi.org/10.1111/jth.14462

3. Garnier-Raveaud S, Usson Y, Cand F, et al (2001) Identification of membrane calcium channels essential for cytoplasmic and nuclear calcium elevations induced by vascular endothelial growth factor in human endothelial cells. Growth Factors Chur Switz 19:35–48. https://doi.org/10.3109/08977190109001074

4. Tu L, Dewachter L, Gore B, et al (2011) Autocrine Fibroblast Growth Factor-2 Signaling Contributes to Altered Endothelial Phenotype in Pulmonary Hypertension. Am J Respir Cell Mol Biol 45:311–322. https://doi.org/10.1165/rcmb.2010-0317OC

5. Bordenave J, Tu L, Berrebeh N, et al (2020) Lineage Tracing Reveals the Dynamic Contribution of Pericytes to the Blood Vessel Remodeling in Pulmonary Hypertension. Arterioscler Thromb Vasc Biol 40:766–782. https://doi.org/10.1161/ATVBAHA.119.313715

6. Sales A, Khodr V, Machillot P, et al (2022) Differential bioactivity of four BMP-family members as function of biomaterial stiffness. Biomaterials 281:121363. https://doi.org/10.1016/j.biomaterials.2022.121363

7. Ricard N, Bidart M, Mallet C, et al (2010) Functional analysis of the BMP9 response of ALK1 mutants from HHT2 patients: a diagnostic tool for novel ACVRL1 mutations. Blood 116:1604–1612. https://doi.org/10.1182/blood-2010-03-276881

8. R Core Team (2022) R: A Language and Environment for Statistical Computing. R Foundation for Statistical Computing, Vienna, Austria

9. Love MI, Huber W, Anders S (2014) Moderated estimation of fold change and dispersion for RNA-seq data with DESeq2. Genome Biol 15:550. https://doi.org/10.1186/s13059-014-0550-8

10. Gu Z (2022) Complex Heatmap Visualization. iMeta. https://doi.org/10.1002/imt2.43

11. Subramanian A, Tamayo P, Mootha VK, et al (2005) Gene set enrichment analysis: A knowledge-based approach for interpreting genome-wide expression profiles. Proc Natl Acad Sci 102:15545–15550. https://doi.org/10.1073/pnas.0506580102

12. Yu G, Wang L-G, Han Y, He Q-Y (2012) clusterProfiler: an R package for comparing biological themes among gene clusters. OMICS J Integr Biol 16:284–287. https://doi.org/10.1089/omi.2011.0118

13. Liberzon A, Subramanian A, Pinchback R, et al (2011) Molecular signatures database (MSigDB) 3.0. Bioinformatics 27:1739–1740. https://doi.org/10.1093/bioinformatics/btr260

**Supplementary figure legends**

***Fig. S1 Characterization of isolated ECFCs from CTL and ALK1-mutated donors***

a Representative phase contrast image of isolated ECFCs displaying the characteristic endothelial cell cobblestone morphology. b Representative immunostaining of the EC marker VE-cadherin on ECFCs. The nuclei were counterstained using Hoechst 33342. c-f Flow cytometric analysis of surface antigens on ECFCs, showing that the isolated cells are positive for the EC markers CD144 (VE-cadherin, c), CD31 (PECAM-1, d) and CD146 (S-Endo 1 antigen, e) but not for the leukocyte common antigen CD45 (f). Isotypic CTL is represented in grey, 3 CTL ECFCs in orange and 2 mutated ECFCs (MUT-H1-H2, Table 1) in blue.

***Fig. S2 Dose response and kinetic of ID1 mRNA expression upon BMP9 stimulation in CTL ECFC***

a Kinetic of *ID1* mRNA expression in response to BMP9 (10 ng/mL), measured by RT-qPCR and normalized to *HPRT* (expressed in fold compared to 0 time point). Data are the mean of three different CTL ECFC donors (H2-4). b *ID1* mRNA expression assessed by RT-qPCR on CTL ECFCs (H2-4) in response to increasing concentrations of BMP9 (from 0.1 to 10 ng/mL) for 18hr and normalized to *HPRT* mRNA expression. Each dot represents one donor. Statistical analysis was performed using Kruskal-Wallis test (*P<0.05).

***Fig. S3 BMP9 and BMP10 induce a similar transcriptomic response in CTL and ALK1-mutated ECFCs***

a, b Venn diagram demonstrating the percentage of common and specific protein coding DEGs induced by BMP9 and BMP10 *vs* NS in CTL ECFCs (a) and in MUT ECFCs (b). c Scatter plot comparing log_2_ fold change (LFC) values of DEGs that are regulated in MUT ECFCs by BMP9 *vs* NS versus those regulated by BMP10 *vs* NS cells. Pearson correlation is reported.

***Fig. S4 BRE luciferase activity of ALK1 mutations identified in ECFCs and HUVECs***

Relative BRE (BMP Response Element) luciferase activity measured in NIH-3T3 cells overexpressing either WT or mutant *ALK1* plasmids identified in *ALK1*-mutated ECFCs or HUVECs that are included in different experiments (p.Gln64X: MUT-H3, p.Arg411Trp: MUT-H4, Thr372HisfsX20: MUT-H5, Cys471Trp: MUT-H6, Table 1). Firefly luciferase activities were normalized to renilla luciferase activities. Data shown are mean ± SEM from of 2 independent experiments.

***Fig. S5 Hierarchical clustering of HMVEC transcriptomic data***

3 CTL and 2 *ALK1*-mutated (MUT-P1-P2) HMVECs were stimulated or not with BMP9 or BMP10 (10 ng/mL) for 18 hours. The experiment was repeated three times after which bulk RNA-seq analysis was performed. Heatmap demonstrating hierarchical clustering based on Euclidian distance applied on all genes across all groups of samples. The samples are separated into 4 clusters: (1) non-stimulated and stimulated MUT-P2 samples, (2) non-stimulated and stimulated MUT-P1 samples, (3) non-stimulated CTL samples and (4) BMP9 and BMP10-stimulated CTL samples. The color code reflects the expression level of a given gene across different samples, with red signifying higher expression and blue signifying lower expression.

**Fig. S6 BM9 and BMP10 induce specific transcriptomic changes in CTL ECFCs versus CTL HMVECs**

Venn diagrams demonstrating the percentage of common and specific protein coding DEGs between CTL ECFCs and CTL HMVECs in response to BMP9 **(A)** or BMP10 **(B)** *vs* NS counterparts.

***Fig. S7* Regulation patterns of hits identified by the two-factor analysis in *ALK1*-mutated HMVECs**

a Heatmap demonstrating the log_2_ fold changes of the 44 protein-coding interaction genes, which were identified by the two-factor analysis as significantly differentially regulated by BMP9 or BMP10 between control and *ALK1*-mutated HMVECs. Genes identified as significantly differentially regulated by both BMP9 and BMP10 are marked by an asterisk and those that were selected for validation by RT-qPCR are marked in blue. b Heatmap demonstrating the fold changes measured by RT-qPCR of the 25 interaction term genes that were selected for individual validation (in blue) in 3 CTL (1-3) and 2 *ALK1*-mutated HMVECs (MUT-P1-2) upon stimulation with BMP9 10ng/mL for 18hr. mRNA expression level of each gene was normalized to *HPRT* mRNA expression and presented as ΔΔCt compared to mean CTL NS. Quantifications shown are mean of 4 independent stimulations. Box color is a reflection of fold change, with white indicating no change, red indicating an upregulation of gene expression and purple indicating a downregulation. Color intensity is proportional to fold change.

***Fig. S8 Cell-surface ALK1 expression in ALK1-mutated HMVECs compared to controls***

Flow cytometric analysis comparing cell-surface ALK1 levels in 3 CTL (1-3) *vs* 2 *ALK1*-mutated HMVECs carrying missense mutations (MUT-P1-2) was carried out. Percentage cell-surface ALK1 expression in mutated HMVECs compared to controls is presented. Data shown are mean ± SEM of 3 independent experiments. Kruskal–Wallis test followed by Dunn’s multiple comparison’s test was used. ns: non-significant

***Fig. S9 RT-qPCR validation of genes identified by two-factor analysis in ALK1-mutated HMVECs, ECFCs and HUVECs***

RT-qPCR quantification of the mRNA expression levels of *JAG2, TNFRSF1B*, *SLC6A6*, *SOX13* and *CEBPG* performed in 3 CTL (1-3), 2 *ALK1*-mutated (MUT-P1-P2) and 3 *BMPR2*-mutated HMVECs (MUT-P3-P5) (a), 3 CTL (H2-4) and 4 *ALK1*-mutated ECFCs (MUT-H1-H4) (b) and 3 CTL (H4-5) and 3 *ALK1-*mutated HUVECs (MUT-H’3-H’5) (c). Target gene expression levels are normalized to *HPRT* mRNA expression presented as ΔΔCt compared to mean CTL NS. Data are mean ± SEM of at least 3 independent stimulations. Two-way Anova followed by Sidak's multiple comparisons test were used for statistical analyses. ns: non-significant, *P<0.05, **P<0.01 and ****P<0.0001 *vs* NS and #P<0.05, ##P<0.01, ###P<0.001 and ####P<0.0001 *vs* CTL.
