# Supplementary figures and images for: Impact of heterozygous ALK1 mutations on the transcriptomic response to BMP9 and BMP10 in endothelial cells from hereditary hemorrhagic telangiectasia and pulmonary arterial hypertension donors
Source: Angiogenesis. 2024 Jan 31;27(2):211–27. doi: 10.1007/s10456-023-09902-8 (PMC11021321; doi:10.1007/s10456-023-09902-8)

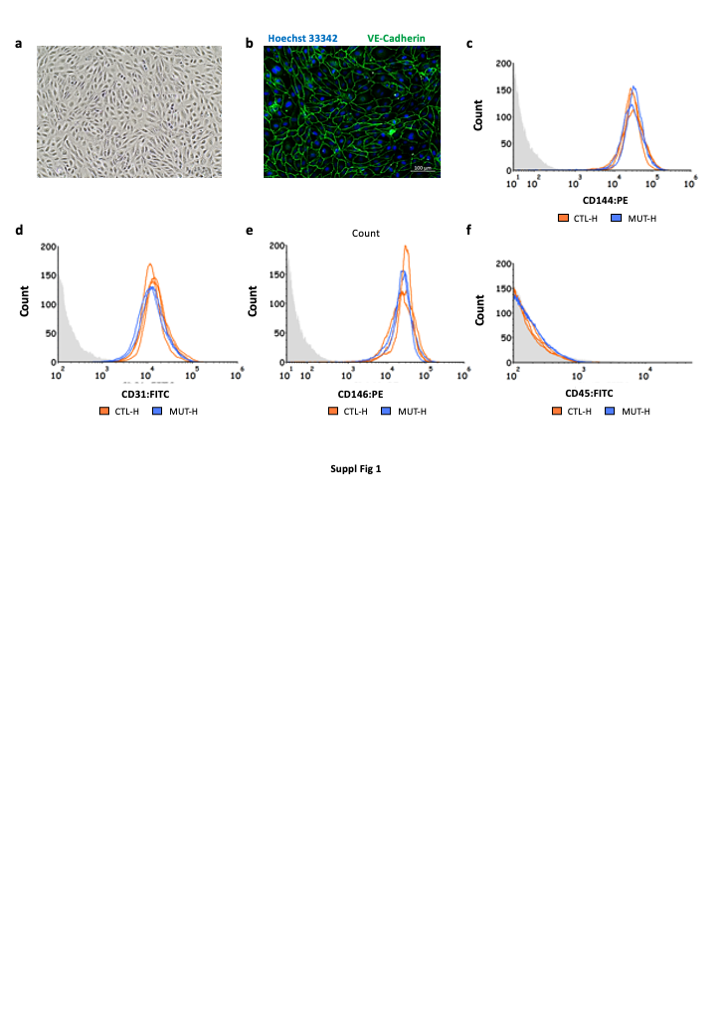

Supplement: Supplementary file 2 — Supplementary file2 (TIFF 2152 kb) [file 10456_2023_9902_MOESM2_ESM.tiff]

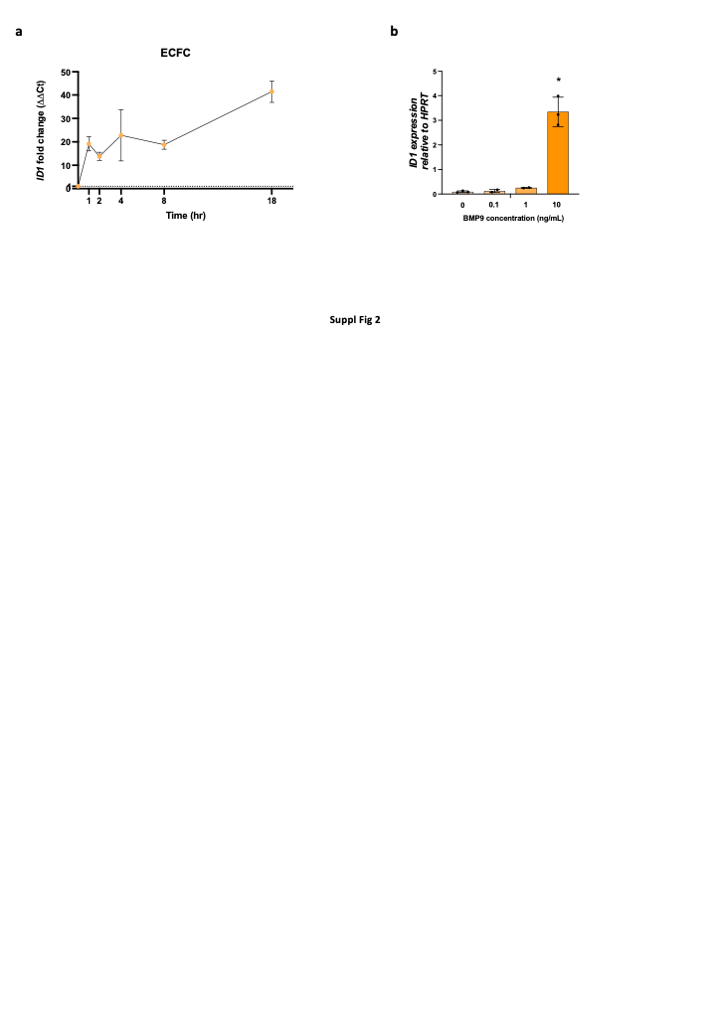

Supplement: Supplementary file 3 — Supplementary file3 (TIFF 2152 kb) [file 10456_2023_9902_MOESM3_ESM.tiff]

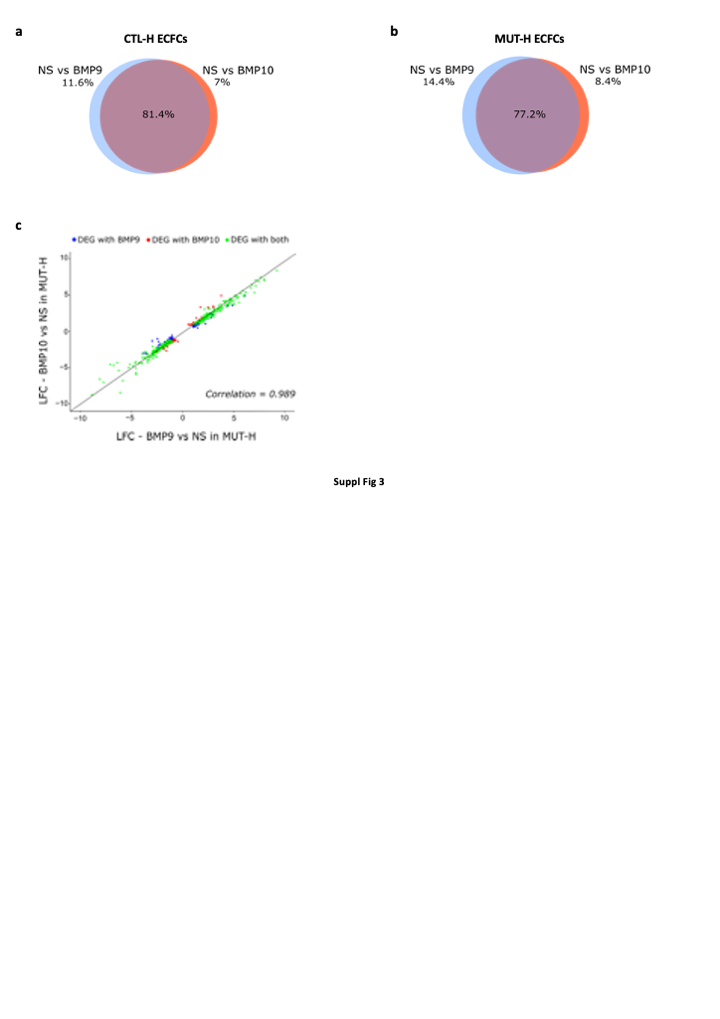

Supplement: Supplementary file 4 — Supplementary file4 (TIFF 2152 kb) [file 10456_2023_9902_MOESM4_ESM.tiff]

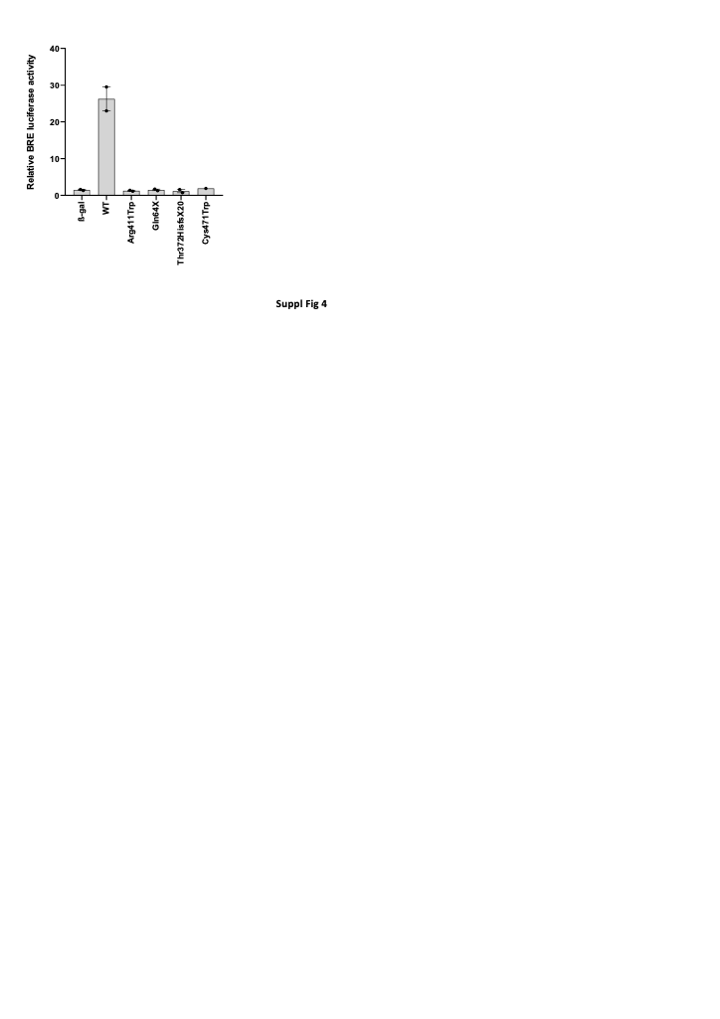

Supplement: Supplementary file 5 — Supplementary file5 (TIFF 2152 kb) [file 10456_2023_9902_MOESM5_ESM.tiff]

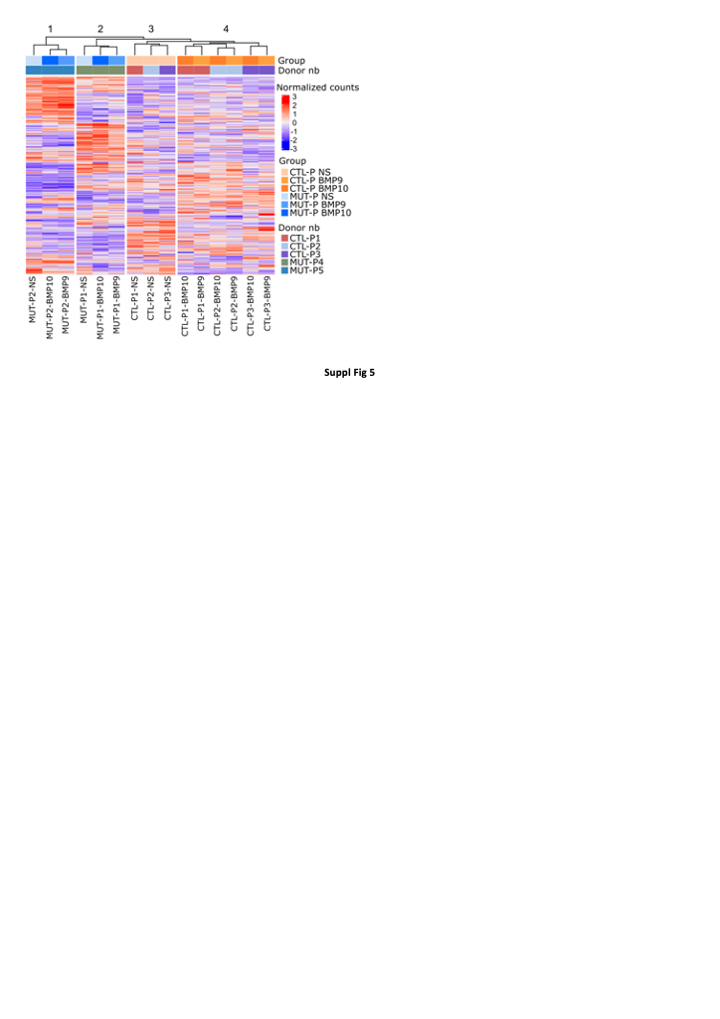

Supplement: Supplementary file 6 — Supplementary file6 (TIFF 2152 kb) [file 10456_2023_9902_MOESM6_ESM.tiff]

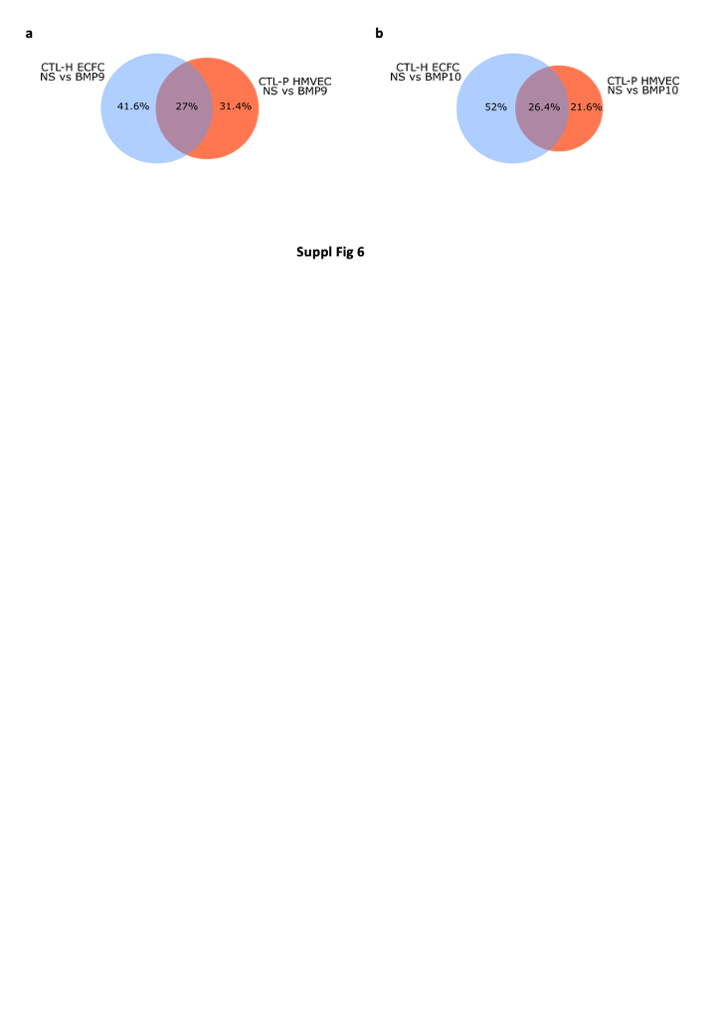

Supplement: Supplementary file 7 — Supplementary file7 (TIFF 2152 kb) [file 10456_2023_9902_MOESM7_ESM.tiff]

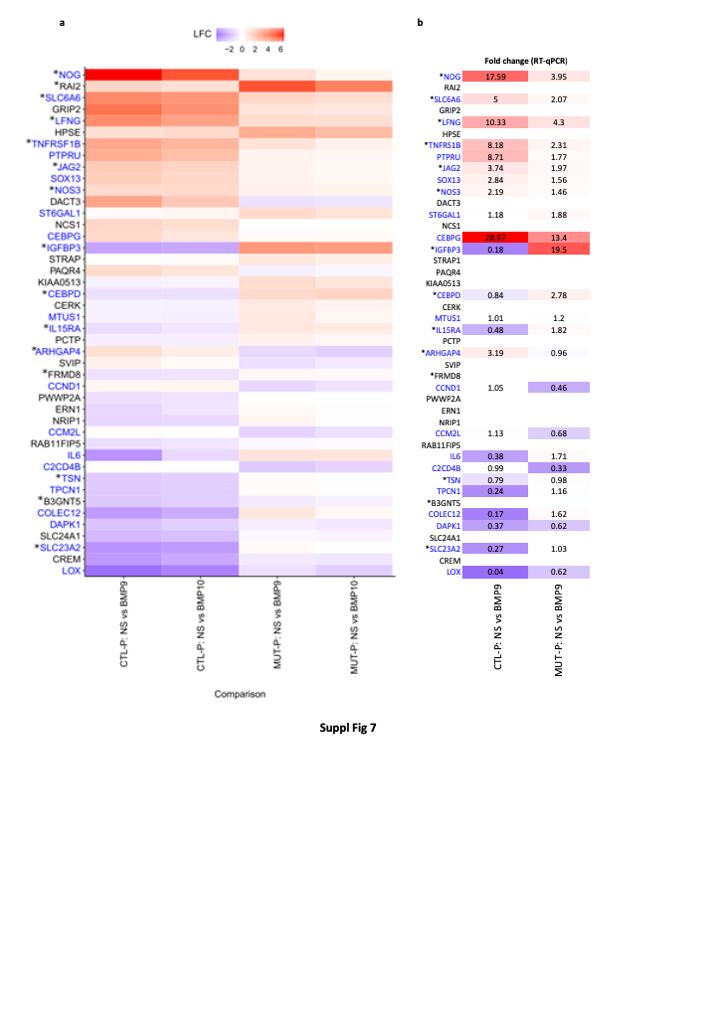

Supplement: Supplementary file 8 — Supplementary file8 (TIFF 2152 kb) [file 10456_2023_9902_MOESM8_ESM.tiff]

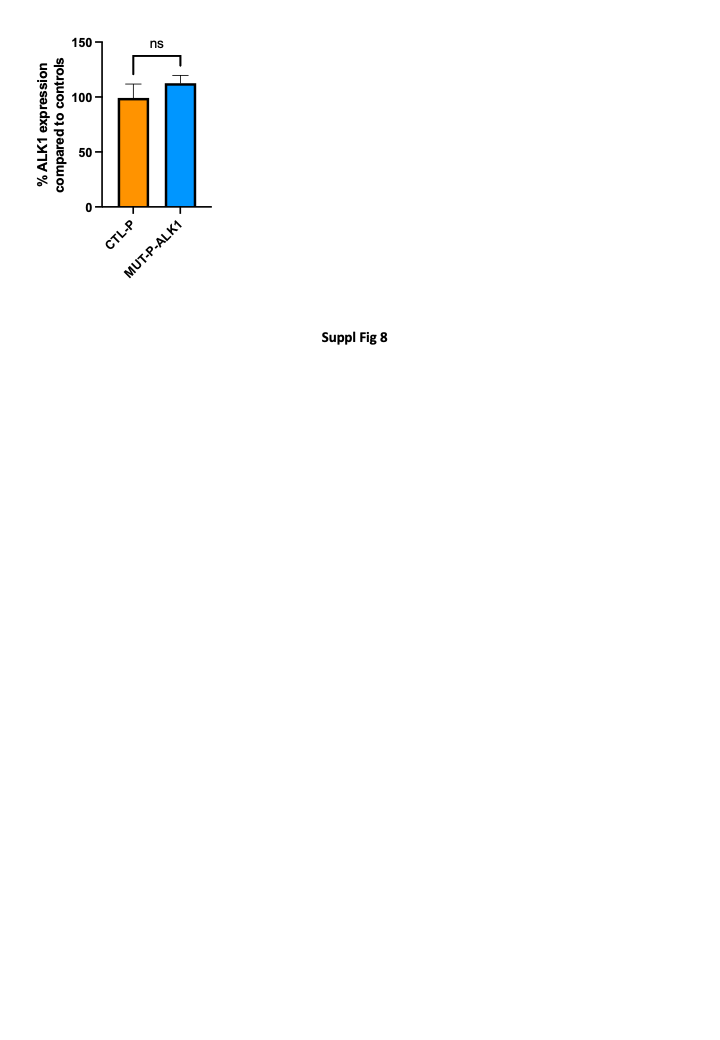

Supplement: Supplementary file 9 — Supplementary file9 (TIFF 2197 kb) [file 10456_2023_9902_MOESM9_ESM.tiff]

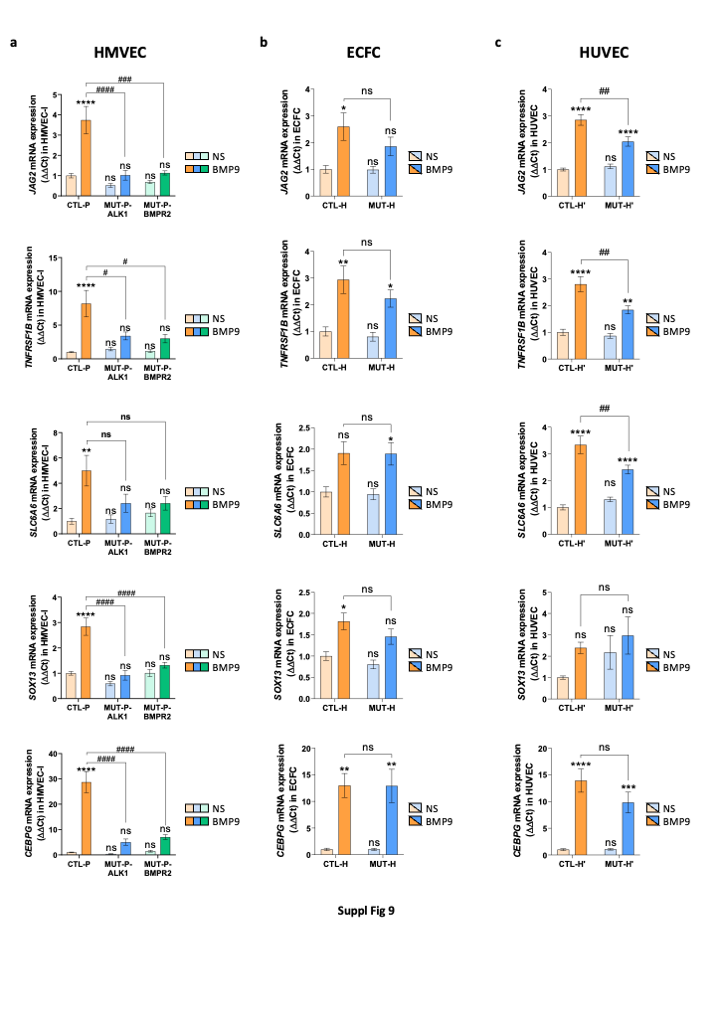

Supplement: Supplementary file 10 — Supplementary file10 (TIFF 2152 kb) [file 10456_2023_9902_MOESM10_ESM.tiff]
