# Supplementary material for: Impact of heterozygous ALK1 mutations on the transcriptomic response to BMP9 and BMP10 in endothelial cells from hereditary hemorrhagic telangiectasia and pulmonary arterial hypertension donors
Source: Angiogenesis. 2024 Jan 31;27(2):211–27. doi: 10.1007/s10456-023-09902-8 (PMC11021321; doi:10.1007/s10456-023-09902-8)
Supplement: Supplementary file 17 — Supplementary file17 (DOCX 13 kb) [file 10456_2023_9902_MOESM17_ESM.docx]

**Suppl Table 1. Primers allowing the generation of mutant *ALK1* constructs by site-directed mutagenesis-associated PCR designed using the QuickChange Primer Design Program (Agilent)**

| Gene | GenBank sequence accession number | mutation | Forward (5’-3’) | Reverse (5’-3’) |
| --- | --- | --- | --- | --- |
| *ACVRL1* | NM_001077401.1 | p.Trp141X | CGTCGGACATGCTACAGGCCCAGGACAC | GTGTCCTGGGCCTGTAGCATGTCCGACG |
|  |  | p.His280Asp | GAGCCGTGCTCGTCGTAGTGCGTGATG | CATCACGCACTACGACGAGCACGGCTC |
|  |  | p.Gly319Arg | GTTTGCCCTGTGTACGGAAGATCTCCACGTG | CACGTGGAGATCTTCCGTACACAGGGCAAAC |
|  |  | p.Arg484Trp | GTGTCTTCTTGATCCACAGCGCGGTGAGTCG | CGACTCACCGCGCTGTGGATCAAGAAGACAC |
|  |  | p.Gln64X | CCCGATGTTCCTAGGGGTGCCTCCC | GGGAGGCACCCCTAGGAACATCGGG |
|  |  | p.Arg411Trp  p.Thr372HisfsX20 | ATTCACGATGGTCCAGCGGGCAATCTCCC | GGGAGATTGCCCGCTGGACCATCGTGAAT |
|  |  | p.Thr372HisfsX20  p.Arg411Gln | GTACCGCTTGGTGCCCCACTCTCGGGT | ACCCGAGAGTGGGGCACCAAGCGGTAC |
|  |  | p.Arg411Gln  p.Cys471Trp | CATTCACGATGGTCTGGCGGGCAATCTCC | GGAGATTGCCCGCCAGACCATCGTGAATG |
|  |  | p.Cys471Trp | GGGTTTGGGTACCACCACTCCCGCATCAT | ATGATGCGGGAGTGGTGGTACCCAAACCC |
