# Supplementary material for: Impact of heterozygous ALK1 mutations on the transcriptomic response to BMP9 and BMP10 in endothelial cells from hereditary hemorrhagic telangiectasia and pulmonary arterial hypertension donors
Source: Angiogenesis. 2024 Jan 31;27(2):211–27. doi: 10.1007/s10456-023-09902-8 (PMC11021321; doi:10.1007/s10456-023-09902-8)
Supplement: Supplementary file 18 — Supplementary file18 (DOCX 15 kb) [file 10456_2023_9902_MOESM18_ESM.docx]

**Suppl Table 2. Primers for quantitative RT-qPCR designed using Primer-Blast on GenBank sequences. All listed primer pairs, except for those of *SLC6A6*, are separated by at least one intron on the corresponding genomic DNA or span an exon-exon junction.**

| Gene | GenBank sequence accession number | Forward (5’-3’) | Reverse (5’-3’) |
| --- | --- | --- | --- |
| *ID1* | NM_181353.3 | CTGCTCTACGACATGAACGGC | TGACGTGCTGGAGAATCTCCA |
| *LFNG*  *ID1* | NM_001040167 | CTTCATCGCTGTCAAGACCAC | GCCTCATCTTCCCCGTCAGT |
| *JAG2* | NM_002226  NM_001066  NM_001066 | GTCGTCATCCCCTTCCAGTTC | ATTCGGGGTGGTATCGTTGT |
| *TNFRSF1B*  *SLC6A6*  *SOX13*  *CEBP* | NM_001066  NM_001134367 | CATGCCGGCTCAGAGAATAC | CTCACAGGAGTCACACACGG |
| *SLC6A6* | NM_001134367 | ATGGGTGATGCTGAGAGCTG | CGCGAAGGAAGCGGTAATTT |
| *SOX13* | NM_005686.3 | AGAAGTGGTGCCAGCCATAG | TCTGCTAGGCTCTCTTGGGT |
| *CEBPG* | NM_001252296.2 | CACTTCGCAGGCATAGTTGG | TCTCCCTTGCCAACACAGAA |
| *ACVRL1* | NM_001077401.1 | CACGGACTGCTTTGAGTCCT | TCTGCTGATCCACACACACC |
| *NOG* | NM_005450.6 | CACTATCTCCACATCCGCCC | GGTCTGGGTGTTCGATGAGG |
| *PTPRU* | NM_005704 | GATGCTGGGAGACCCCAATG | GCTATGAAGTGGTTTGACCTGT |
| *NOS3* | NM_000603.4 | GTGGCTGGTACATGAGCACT | GTCTTTCCACAGGGACGAGG |
| *IGFPB3*  *CEBPD*  *MTSU1*  *Il15RA* | NM_001013398.2 | CGCCAGGAAATGCTAGTGAG | AACTTGGGATCAGACACCCG |
| *CEBPD* | NM_005195 | TGGGACATAGGAGCGCAAAG | ACACGTTTAGCTTCTCTCGCA |
| *MTUS1* | NM_001001924.3 | TGAGAAACTGCATCAACAGGAC | ATTGCCATGTGCTTGTCCATCC |
| *IL15RA* | NM_001351095 | TCCAGGGAGCGGTACATTTG | TGAAGCTGCGGGCTTAATG |
| *ARHGAP4* | NM_001164741.2 | TGCGCTTTGACTACCACCC | TGTCTCAATGGTCTGTCGGT |
| *CCND1* | NM_053056.3 | CCAAAATGCCAGAGGCGGA | AGGGCGGATTGGAAATGAAC |
| *CCM2L* | NM_001365692 | ACACATCCACACCTGAACGG | GGCATACGTCCCATCTGTGT |
| *Il6* | NM_000600.5 GI:1531243779. | GAGTAGTGAGGAACAAGCCAGA | GTTGGGTCAGGGGTGGTTATT |
| *C2CD4B*  *TSN*  *T* | NM_001007595.3 | TGAAGAAGAGAGAAAGGCGCAC | GTTTCTCGAGGAGCCGCAT |
| *TSN* | NM_004622 | TGAGCCAGATCGGGAGAAA | AGTAGTCTCCAGCAGTCACG |
| *TPCN1* | NM_001351346.2 | GAAAGGGAGCTCAAACCAGAGA | CCAAGGTCAGGATGAGCGG |
| *COLEC12* | NM_130386.3 | CAGAGGAGGAGGAGGTGCAATC | ACATTTGGTACATTGTGTTCCTTCC |
| *DAPK1* | NM_004938 | TTCAGGCAGGAAAACGTGGA | GGCATTTCTTCACAACCGCA |
| *SLC23A2* | NM_203327 | GGGGGCTGTGTGGCTTTTAT | GCCCACACCCTTCTTCCATT |
| *LOX* | NM_002317.7 | CAC TGG CTA CTT CCA GTA CG | ACATCTGCCCTGTATGCTGT |
